# Integration of HIV testing services into family planning services: a systematic review

**DOI:** 10.1186/s12978-019-0714-9

**Published:** 2019-05-29

**Authors:** Manjulaa Narasimhan, Ping Teresa Yeh, Sabina Haberlen, Charlotte E. Warren, Caitlin E. Kennedy

**Affiliations:** 1 Department of Reproductive Health and Research and UNDP/UNFPA/UNICEF/WHO/World Bank Special Programme, World Health Organization, Geneva, Switzerland; 2 Department of International Health, Johns Hopkins Bloomberg School of Public Health, Baltimore, MD USA; 3 Department of Epidemiology, Johns Hopkins Bloomberg School of Public Health, Baltimore, MD USA; 4 Population Council, Washington, DC USA

**Keywords:** Service integration, Family planning, HIV, Sexual and reproductive health, Access

## Abstract

**Background:**

Despite significant interest in integrating sexual and reproductive health (SRH) services into HIV services, less attention has been paid to linkages in the other direction. Where women and girls are at risk of HIV, offering HIV testing services (HTS) during their visits to family planning (FP) services offers important opportunities to address both HIV and unwanted pregnancy needs simultaneously.

**Methods:**

We conducted a systematic review of studies comparing FP services with integrated HTS to those without integrated HTS or with a lower level of integration (e.g., referral versus on-site services), on the following outcomes: uptake/counseling/offer of HTS, new cases of HIV identified, linkage to HIV care and treatment, dual method use, client satisfaction and service quality, and provider knowledge and attitudes about integrating HTS. We searched three online databases and included studies published in a peer-reviewed journal prior to the search date of June 20, 2017.

**Results:**

Of 530 citations identified, six studies ultimately met the inclusion criteria. Three studies were conducted in Kenya, and one each in Uganda, Swaziland, and the USA. Most were in FP clinics. Three were from the Integra Initiative. Overall rigor was moderate, with one cluster-randomized trial. HTS uptake was generally higher with integrated sites versus comparison or pre-integration sites, including in adjusted analyses, though outcomes varied slightly across studies. One study found that women at integrated sites were more likely to have high satisfaction with services, but experienced longer waiting times. One study found a small increase in HIV seropositivity among female patients testing after full integration, compared to a dedicated HIV tester. No studies comparatively measured linkage to HIV care and treatment, dual method use, or provider knowledge/attitudes.

**Conclusions:**

Global progress and success for reaching SRH and HIV targets depends on progress in sub-Saharan Africa, where women bear a high burden of both unintended pregnancy and sexually transmitted infections, including HIV. While the evidence base is limited, it suggests that integration of HTS into FP services is feasible and has potential for positive joint outcomes. The success and scale-up of this approach will depend on population needs and health system factors.

**Electronic supplementary material:**

The online version of this article (10.1186/s12978-019-0714-9) contains supplementary material, which is available to authorized users.

## Background

Global scale-up of antiretroviral therapy has been the primary contributor to a 48% decline in deaths from AIDS-related causes, but AIDS-related illnesses still remain a leading cause of death among women of reproductive age (15–49 years) particularly in sub-Saharan Africa [1]. Where women and girls are at risk of HIV, offering HIV testing during their visits to family planning (FP) services offers important opportunities to address HIV and unwanted pregnancy simultaneously. The World Health Organization (WHO) global reproductive health strategy outlines a comprehensive approach to sexual and reproductive health (SRH) that includes HIV, [2] and integrating HIV testing services into FP services can contribute to achieving joint health and human rights outcomes and accelerate progress towards comprehensive SRH and rights [3, 4].

Attention has been given to integrating FP services into HIV testing, care, and treatment services [5–7] and provision of FP counseling for women living with HIV, [8–10] but there has been comparatively less attention to integrating HTS into FP sites, even though there is evidence that routine, opt-out HIV testing integrated into FP clinics can potentially increase rates of testing acceptance, receipt of test results, and HIV-positive diagnoses among adolescents and young adults [11]. Sexually active FP clients, in particular those living in high HIV prevalence settings or engaging in behaviors that put them at higher risk of HIV, may also benefit from HTS. A 2009 systematic review of multi-directional linkages between family planning and HIV services identified two studies that provided HTS to clients of family planning clinics [12]. One study from the Dominican Republic added HTS and HIV treatment to existing FP services provided at a clinic; [13] another study from South Africa compared on-site provision of HTS to FP clinic clients with off-site HTS referral [14]. However, neither was published as a peer-reviewed article. Another comprehensive review in 2009 examined the impact of integrating any component of STI or HIV prevention, care and treatment into FP consultations. The evidence demonstrated the potential of integrating services such as client satisfaction and reduce clinic-based HIV stigma. For example, integration of SRH and HIV services in Botswana demonstrated high (82.7%) client satisfaction with services, especially because clients felt integration reduced the number of trips to the health facility [15]. Nevertheless, it was apparent that providers frequently missed opportunities to integrate care as well as other programmatic challenges to maintain quality of care [16]. Apart from a Cochrane review in 2012 that looked at bidirectional integration of HIV/AIDS services with maternal, neonatal and child health, nutrition, and FP services, [17] there have not been any more recent systematic reviews specific to integrating HTS into FP services, which remains an important programmatic gap in providing these services.

This paper examines the evidence for the integration of HTS into FP services. We hoped to identify what models of integrating such services have been evaluated, along with their positive and negative outcomes.

## Methods

### Definitions

For the purposes of this review, we used the following definitions:

- **Linkages** refer to bi-directional synergies in policy, systems, and services between sexual and reproductive health and rights and HIV. It refers to a broader human rights-based approach, of which SRH service integration is a subset [18, 19].
- **Integration** refers to the service delivery level and can be understood as joining operational programmes to ensure effective outcomes through many modalities (such as multi-tasked providers, referral, and one-stop shop services under one roof) [18].

WHO, UNFPA, IPPF, and UNAIDS developed a framework for SRH/HIV linkages and defined integration at the service delivery level as “different kinds of MNCHN [maternal, neonatal, and child health and nutrition] and HIV services or operational programs joined together to ensure and perhaps maximize collective outcomes [20].” For the purposes of this review, we use this definition of integration and focus on the service delivery level, though we recognize the many other existing definitions.

- **HIV Testing Services (HTS)** is defined by WHO as “the full range of services that should be provided together with HIV testing – counselling (pre-test information and post-test counselling); linkage to appropriate HIV prevention, treatment and care services and other clinical and support services; and coordination with laboratory services to support quality assurance and the delivery of correct results [21].”
- **Family planning and Contraception** has direct health benefits, such as prevention of unintended pregnancy and, subsequently, decreased maternal mortality and morbidity [22]. Providing family planning services can include various contraceptive methods as well as meeting the fertility needs of individuals, pregnancy testing and counseling, conception planning, basic infertility services, preconception health services, and the screening and treatment of sexually transmitted infections, and postpartum family planning.

### PICO question

**PICO:** Should HTS be integrated into FP services?

**P:** FP service clients

**I:** HTS integrated with FP services

**C:** FP services without integrated HTS, or with a lower level of integration (e.g., referral instead of on-site services)

**O:** (1) uptake of, counseling for, or offer of HTS, (2) new cases of HIV identified, (3) linkage to HIV care and treatment, (4) dual method use, (5) client satisfaction and service quality, (6) provider knowledge and attitudes about integrating HTS

### Search and screening process

To be included in the review, an article had to meet the following inclusion criteria:

1. Comparative study examining FP service users (with or without their partners) who received FP services at sites with integrated HTS compared with FP clients who received FP services without integrated HTS, or with a lower level of integration, on one or more of the key outcomes outlined in the PICO question above.
2. Published or accepted for publication in a peer-reviewed journal prior to the search date of June 20, 2017.

FP services were considered any site where FP services are routinely provided, including stand-alone FP clinics, mobile services, or sites that provide FP. We included postpartum FP services; however, we excluded antenatal care services, because there has already been significant consideration of integration of HIV testing into antenatal care as part of prevention of vertical transmission programs.

Studies comparing opt-in versus opt-out HIV testing in FP services were not included, as these studies are considered two different approaches to HIV testing rather than different models of integration. There were no restrictions by language or geographic area.

Three electronic databases were searched through June 20, 2017: PubMed, CINAHL (Cumulative Index to Nursing and Allied Health Literature), and EMBASE. The following terms were used to search PubMed and adapted for the controlled vocabulary of the other databases: (“HIV test*” [tiab] OR “HIV counselling and testing” [tiab] OR “HIV counseling and testing” [tiab]) AND (“family planning services”[mesh] OR contracepti* [tiab] OR “family planning” [tiab] OR “postpartum family planning” [tiab]).

Secondary reference searching was conducted on all studies included in the review and a related previous review [12]. We also contacted authors of ongoing studies related to this topic, such as the Integra Initiative, [23] to identify additional articles.

Titles, abstracts, citation information, and descriptor terms of citations identified through the search strategy were initially screened by one member of the study staff. Remaining abstracts were screened in duplicate by two reviewers working independently, with adjudication of differences by a senior member of the study team. Two independent reviewers assessed full-text articles for eligibility to determine final study selection.

### Data extraction and analysis

Data were extracted using standardized forms. The following information, adapted from the previous review, [12] was gathered from each included study: Study citation, country, setting (urban/rural), setting (type of clinic/service), target group, years of program, years of evaluation, name of program, intervention, format of integration (on-site, referral, etc.), cost of services, study design, unit of analysis, sample size, sample age, sample gender, length of follow-up, reported outcomes and results.

Study rigor was assessed using on a nine-item tool with items for: (1) Study design including pre/post intervention data, (2) Study design including control or comparison group, (3) Study design including cohort, (4) Comparison groups equivalent at baseline on socio-demographics, (5) Comparison groups equivalent at baseline on outcome measures, (6) Random assignment (group or individual) to the intervention, (7) Participants randomly selected for assessment, (8) Control for potential confounders, (9) Follow-up rate > =75%. This scale is based on the eight-item rigor assessment scale previously developed for systematic reviews of HIV behavioral interventions [24].

Data were analyzed descriptively. Due to a lack of similar studies with combinable outcomes, meta-analysis was not possible.

## Results

### Search results

**Fig. 1:**
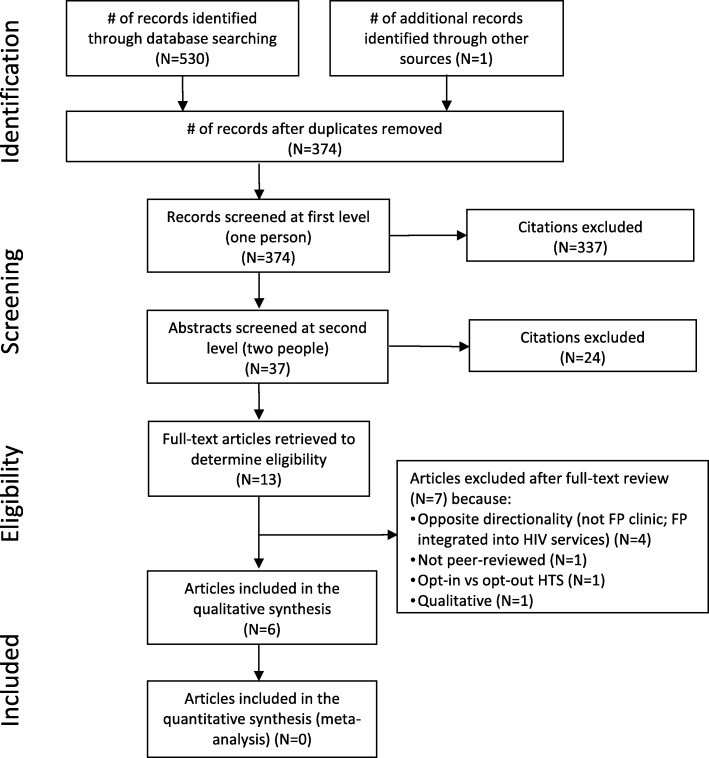
PRISMA flow chart showing disposition of search results

Initial database searching yielded 530 citations, with one citation identified through other means; 374 citations remained after removing duplicates (Fig. 1). Initial screening excluded 337 citations and secondary screening excluded 24 for not meeting the inclusion criteria. After thoroughly reviewing and discussing the remaining 13 articles, seven more were excluded. Ultimately, six articles met the criteria for inclusion [25–30]. Table 1 presents descriptions of the study settings, methods, and outcomes. Table 2 presents an assessment of study rigor. Table 3 presents a summary of key outcome findings.

**Table 1:** Descriptions of included studies

Study	Setting	Intervention	Study Design	Sample size
Birdthistle et al., 2014 [25]	Location: Swaziland / urban, rural, and peri-urban / MCH units of public sector (government) health facilities Target group: Female clients	Years of program: 2009–2012 Years of evaluation: 2009–2012 Name: Integra Initiative Intervention: Activities and resources to strengthen integration of HIV services into postnatal care: (1) Training package to facilitate mentoring of front-line health providers (2) job aids to promote integration (3) ongoing support to discuss role clarification, organizational change, referral/linkages, and management of service statistics Format: On-site referrals	Study design: Group non-randomized trial Selection of sites: Purposive Selection of participants: Consecutive	Sample size: 3261 female clients were tracked in 2009, 2086 in 2010 and 2916 in 2012 Age: N/A Gender: Female Follow-up: N/A
Brunie et al., 2016 [26]	Location: Uganda / NR / health center Target group: Clients	Years of program: 2012–2013 Years of evaluation: 2013 Name: N/A Intervention: Village health teams trained to offer HTC along with family planning and linked to health centers for supervision, commodity supply, and referral management Format: On-site testing, referral to health clinic	Study design: Group randomized trial Selection of sites: Purposive Selection of participants: Systematic (every n^th^ client)	Sample size: 256 clients Age: Mean (SE): intervention group 31.02 (0.40), control group 30.73 (1.14) Gender: N/A Follow-up: N/A
Church et al., 2017 [27]	Location: Kenya / urban, rural, and peri-urban / health centers and hospitals Target group: Female family planning clients	Years of program: 2009–2012 Years of evaluation: 2010–2012 Name: Integra Initiative Intervention: SRH/HIV integration added the following services to standard FP service delivery: discussion of fertility desires, condom promotion/provision, STI/HIV risk assessment, HIV status check, HTS provision, cervical cancer screening, pre-HIV treatment services, and/or referral to HIV treatment unit for HIV+ clients Format: On-site testing, pre-HIV treatment services and/or referral to HIV treatment clinic	Study design: Group non-randomized trial Selection of sites: Purposive Selection of participants: Consecutive	Sample size: 882 Age: 15–49 years Gender: Female Follow-up: Original recruitment 1958: excluded 245 known to be HIV+, 745 without complete cohort data history, 86 missing complete data on all potentially confounding variables
Criniti et al., 2011 [28]	Location: USA / urban / Title X-funded FP clinic Target group: Female family planning clients	Years of program: 2007–2009 Years of evaluation: 2007–2009 Name: N/A Intervention: Capacity building for clinic medical staff to perform routine non-targeted rapid HIV testing Format: On-site testing and referral to HIV-specialized prenatal clinic within FP center	Study design: Retrospective cohort study Selection of site: NR Selection of participants: Consecutive	Sample size: NR (overall sample of client records NR; patient population of approximately 9000/year) Age: 15–49 years Gender: Female Follow-up: N/A
Kimani et al., 2015 [29]	Location: Kenya / rural and peri-urban / public health facilities (health centers, dispensaries, hospitals) Target group: Postpartum women 15–49 years old	Years of program: 2010–2012 Years of evaluation: 2010–2012 Name: Integra Initiative Intervention: Integrated HIV and FP services into postnatal care compared to standalone services Format: On-site testing and counseling	Study design: Group non-randomized trial Selection of sites: Purposive Selection of participants: NR	Sample size: 1693 (815 intervention, 878 comparison) ge: 15–49 years Gender: Female Follow-up: 71% (573 intervention, 631 comparison)
Liambila et al., 2009 [30]	Location: Kenya / NR / Family planning with provider-initiated testing and counseling (integrated HTS) public-sector hospitals, health centers, and dispensaries Target group: Female family planning clients	Years of program: 2005–2007 Years of evaluation: 2006–2007 Name: N/A Intervention: Family planning with provider-initiated testing and counseling (integrated HTS) Format: On-site testing and counseling	Study design: Group non-randomized trial Selection of sites: Purposive Selection of participants: Consecutive	Sample size: 1058 Age: Most were around 30 years old Gender: Female Follow-up: N/A

*HTS* HIV testing services, *FP* Family planning, *MCH* Maternal child health, *STI* Sexually transmitted infection, *SE* Standard error, *NR* Not reported, *N/A* Not applicable

**Table 2:** Study rigor

Study	Study design includes pre/post intervention data	Study design includes control or comparison group	Study design includes cohort	Comparison groups equivalent at baseline on socio-demographics	Comparison groups equivalent at baseline on outcome measures	Random assignment (group or individual) to the intervention	Participants randomly selected for assessment	Control for potential confounders	Follow-up rate > = 75%
Birdthistle et al., 2014 [25]	Yes	Yes	No	No	No	No	Yes^a^	Yes^b^	NA
Brunie et al., 2016 [26]	No	Yes	No	Yes	NA	Yes	Yes	No	NA
Church et al., 2017 [27]	No	Yes	Yes	No	No	No	Yes^a^	Yes	No
Criniti et al., 2011 [28]	Yes	No	No	NA	NA	No	Yes^a^	No	NA
Kimani et al., 2015 [29]	Yes	Yes	Yes	Yes	No	No	No	Yes	No
Liambila et al., 2009 [30]	Yes	Yes	No	No	No	No	Yes^a^	No	N/A

^a^Consecutive sampling / census selection

^b^For limited confounders: facility client load, baseline integration, rural/urban

**Table 3:** Summary of key outcome findings

Study	Outcome category from PICO question				
	1) Uptake of, counseling for, or offer of HIV testing services				5) Client satisfaction / perceptions of service quality
Birdthistle et al., 2014 [25]		Control sites (*n* = 4)	Intervention sites (*n* = 4)		Not measured
	Proportion of visits where women received HIV counseling and testing				
	2009	5–30%	3–27%		
	2010	2–14%	8–16%		
	2012	6–58%	3–15%		
	Proportion of visits where women received HIV/STI services and MCH services				
	2009	11–49%	9–33%		
	2010	3–27%	2–21%		
	2012	14–44%	10–17%		
Brunie et al., 2016 [26]		Control group (*n* = 119)	Intervention (*n* = 137)	*p*-value	Not measured
	Ever tested for HIV, n (%)	113 (94.96%)	136 (99.27%)	0.002	
	Number of tests in the past 12 months, n (%)			0.043	
	0	22 (18.49%)	10 (7.35%)		
	1	20 (16.81%)	20 (14.71%)		
	2	31 (26.05%)	28 (20.59%)		
	3	34 (28.57%)	44 (32.35%)		
	> 4	12 (10.08%)	34 (25.00%)		
Church et al., 2017 [27]		Intervention group (*n* = 439)	Comparison group (*n* = 443)		• Women at the intervention sites were more likely to have high satisfaction with services (30% versus 27%) • Women at the intervention sites were more likely to wait longer than 30 min for services (57%, versus 0.2%) • Women at the intervention sites were less likely to have paid fees for services (83% versus 93%).
	Proportion who reported receiving an HIV test since last interview				
	R0 (immediately post-intervention)	8.4	47.6		
	R1 (+ 6 months)	44.7	51.5		
	R2 (+ 18 months)	64.0	66.4		
	R3 (+ 24 months)	71.8	60.7		
	Percent of women achieving HIV testing goals (two-test minimum, one test per year) over the two-year cohort, by different exposure groups • More women in the HIV comparison group (73%) met the HIV testing goal compared to the intervention group (65%) (*p* < 0.05). • Women who received integrated services at baseline, regardless of design group, were more likely to receive the two-test minimum after r0 (71%) compared to those who did not (61%) (*p* < 0.01). • Women with highest cumulative exposure to integrated services were more likely to have received the testing requirement (77%) versus the medium score group (71%) and the low score group (60%) (*p* < 0.001).				
Criniti et al., 2011 [28]		Prior to HIV rapid testing (before 2003)	Designated HIV tester (2003–2007)	Full integration into clinic flow (2007–2009)	Not measured
	Testing acceptance rate	Unavailable	76%	89%	
	Patients with a documented HIV test in medical chart from previous 12 months	34%	65%	71%	
	Average tests performed per month	Unavailable	70	87.9	
Kimani et al., 2015 [29]		Control group n/N (%)	Intervention group n/N (%)		Not measured
	Uptake of Provider-initiated testing and counseling				
	Baseline	87/878 (9.9)	125/815 (15.3)		
	15-month follow-up	104/631 (29.6)	157/573 (46.6)		
	aOR for intervention site compared to control: 1.6, (95% CI: 1.2–2.2) (*p* < 0.01)				
Liambila et al., 2009 [30]		Testing model % (N)	Referral model % (N)		Not measured
	Proportion of new clients being tested after introducing the intervention				
	New clients offered HIV test*	74 (27)	34 (50)		
	If offered, new clients choosing HIV test	50 (20)	65 (17)		
	Proportion of all new clients being tested*	37 (27)	22 (50)		
	Proportion of revisit clients being tested				
	Revisit clients offered HIV test*	56 (183)	27 (259)		
	If offered, revisit clients choosing HIV test	61 (103)	72 (69)		
	Proportion of all revisit clients being tested*	34 (183)	19 (259)		
	Proportion of all clients tested				
	Proportion of all new and revisit clients being tested*	35 (210)	20 (309)		

Note: this table only includes outcome data that met the PICO question by comparing HIV testing services integrated into family planning services to non-integrated services. None of the studies reported PICO outcomes #2) new cases of HIV identified, #3) linkages to HIV care and treatment, #4) dual method use, and #6) provider knowledge and attitudes about integrating HTS

*significant at *p* < 0.01

### Study descriptions

Five of the six studies were from sub-Saharan Africa (Table 1). Three were conducted in Kenya, [27, 29, 30] while one each was conducted in Uganda, [26] Swaziland, [25] and the USA [28]. Three were conducted as part of the Integra Initiative [25, 27, 29]. Most studies were conducted in FP clinics, though two were in a postnatal setting [25, 29]. Most services were provided at static clinic sites, with on-site testing and counseling and referral to HIV care and treatment services for people whose HIV test results came back positive. However, one study evaluated the effectiveness of village health teams (VHT) trained to offer HTS along with FP; the VHT were linked to health centers for supervision, commodity supply, and referral management [26]. All except one focused on female client populations.

Overall rigor was moderate (Table 2). There was one group randomized trial and four group non-randomized trials; the remaining study was a retrospective cohort. Most studies purposively selected facilities and then consecutively sampled clients within those facilities. Three studies controlled for potential confounding factors in their analyses.

### Study findings: uptake of, counseling for, or offer of HTS

HTS uptake, measured in a variety of different ways, was the most common outcome, measured in all six studies. HTS uptake was generally higher in integrated sites compared with comparison or pre-integration sites, including in adjusted analyses, though there were some differences in outcomes across studies.

In Uganda, the cluster-randomized trial evaluating VHTs found that intervention group participants were significantly more likely than control participants to report having ever tested for HIV (99.27% vs. 94.96%, *p* = 0.002) and having more HIV tests in the past 12 months (*p* = 0.043) [26].

In Kenya, integrated family planning with provider-initiated testing and counseling (PITC) in public-sector hospitals, health centers, and dispensaries – was associated with significantly higher percentages of being offered an HIV test among both new clients (74% vs. 34%) and revisit clients (56% vs. 27%), compared to a referral model [30]. While there was a non-significantly higher proportion of both new and revisit clients who refused the test when offered, there was still a significantly higher proportion of clients being tested overall (35% vs. 20%), and this was significant among both new (37% vs. 22%) and revisit (34% vs. 19%) clients.

In the United States, an urban Title X-funded FP clinic transitioned from using a designated HIV counselor for targeted HTS to a model using clinic staff to provide integrated, routine, non-targeted, rapid HTS as standard of care within the FP center (full integration) [28]. Testing acceptance rates increased from 76% during the designated HIV tester period to 89% under full integration; similarly, the percentage of patients with a documented HIV test in their medical charts in the previous 12 months increased from 34% prior to integrating any HTS to 65% in the designated HIV tester period to 71% under full integration.

Two studies, both by the Integra Initiative, examined postnatal care settings. In Kenya, where integrated HIV and FP services into postnatal care were compared to standalone services, the odds of PITC uptake were higher in the intervention sites compared to the comparison sites (aOR = 1.6, *p* < 0.01, 95% CI: 1.2–2.2) [29]. In Swaziland, activities and resources to strengthen integration of HIV services into postnatal care services included a training package to facilitate mentoring of front-line health providers, job aids to promote integration, and ongoing support to discuss role clarification, organizational change, referral/linkages, and management of service statistics [25]. HIV counseling received increased in two intervention and two comparison facilities and fell in one intervention facility and two comparison facilities; one intervention facility did not show significant change in this outcome. However, the study also noted that they could not specify which sites actually integrated which services.

In the final study conducted by the Integra Initiative in Kenya, SRH/HIV integration added the following services to standard FP service delivery: discussion of fertility desires, condom promotion/provision, STI/HIV risk assessment, HIV status check, HTS provision, cervical cancer screening, pre-HIV treatment services, and/or referral to HIV treatment unit for HIV-positive clients [27]. The proportion of clients who reported receiving an HIV test since the last interview increased from 8.4% at baseline to 71.8% at 24 month follow-up in the intervention group compared to 47.6 to 60.7% for the control group. The percent of women achieving what the study considered HIV testing goals (two-test minimum, one test per year) over the two-year cohort, was actually higher in the comparison group than in the intervention group (*p* < 0.05). However, among those who received integrated services at baseline, regardless of design group (71%), compared to those who did not (61%) (*p* < 0.01). Further, women with the highest cumulative exposure to integrated services were more likely to have received the testing requirement (77%) versus the medium score group (71%) and the low score group (60%) (*p* < 0.001).

### Study findings: new cases of HIV and linkage to HIV care and treatment

No studies comparatively measured new cases of HIV identified (yield) or linkage to HIV care and treatment. However, one study from the United States did measure seropositivity (not further specifying whether these were new or already diagnosed cases) [28]. While seropositivity rates were not available for the period prior to rapid testing, < 0.5% all patients tested HIV-positive during the period of the designated tester, while 0.7% of patients (0.6% of women, 10% of men) tested HIV-positive during the period of full integration. While no comparative data were presented for linkage to HIV care and treatment, two studies did note the total number of clients testing positive (range of 3–16 individuals) and noted that all were linked to medical care [26, 28].

### Study findings: dual method use

No studies provided comparative data on dual method use.

### Study findings: client satisfaction and service quality

Only one study reported comparative (pre-post or multi-arm) indicators on client satisfaction and perceptions of service quality around the integration of HTS into FP services. This study from the Integra Initiative in Kenya used a mean score based on Likert scales on “overall service rating, costs, waiting time, availability of drugs and supplies, possibility of receiving other services at the same time, opening times, provider friendliness, doctor/nurse availability, providers listened, client could ask questions [27].” Women at the intervention sites were more likely to have high satisfaction with services (30% versus 27%), but waited longer than 30 min (57%, versus 0.2%) and were less likely to have paid fees for services (83% versus 93%).

The cluster-randomized trial of VHTs in Uganda did include non-comparative measures of client satisfaction and service quality within the intervention group only [26]. Over 95% of clients tested by a VHT responded positively to questions on satisfaction with interpersonal relationships and with information and services received. The vast majority (99.1%) also said that they trusted the VHT with private information. All clients tested by the VHT who were HIV-negative intended to get tested in the future, and 93.5% said they preferred a VHT for their next test. VHT’s average composite knowledge score was 5.1 out of 7 possible points, with 81.6% of VHTs scoring at least 5; the main knowledge gaps were recommended frequency for repeat testing among HIV-negative clients and safety measures. Of 34 VHTs who participated in quality assurance, 85.3% passed with 100% concordance with the reference laboratory. Client reports suggested that no clients tested by a VHT reported any problems with finger-pricking procedures. The majority of clients reported that VHTs had provided key HTS counseling messages.

### Study findings: provider knowledge and attitudes about integrating HTS

There were no comparative outcomes presented on provider knowledge and attitudes about integrating HTS. However, one study from the United States measured provider attitudes 6 months after the intervention with a 70% response rate [28]. Using a Likert scale, 100% of respondents rated offering routine HIV screening to all patients as “very important”; 78% rated the integration of HIV testing as “very” or “somewhat successful”; and 56% reported having performed HIV testing in the clinic. All staff rated having on-site support from experienced HIV counselors as “most helpful”.

## Discussion

While the evidence base is limited, existing studies indicate that integration of HTS into FP services is feasible and has the potential for positive outcomes. All six papers described here measured HTS uptake as the main outcome. However, the other five outcome measures we selected a priori (new cases of HIV identified; linkage to HIV care and treatment; dual method use; client satisfaction and service quality; provider knowledge and attitudes about integrating HTS) had limited comparative measurement.

It is a significant gap in the literature that no studies provided comparative measures of new cases of HIV identified and dual method use, and few provided comparative measures of linkages to care and treatment, client satisfaction and service quality, or provider knowledge and attitudes about integrating HTS. The goal of HIV testing is to identify people living with HIV who have not yet received a diagnosis, with the next step of linking them to HIV services; and for those who test HIV negative to have access to prevention interventions in order for them to remain HIV-free. Therefore, information on case yield and strengthening linkages to care is critical, and may be particularly important in identifying where integration of HTS into FP services makes sense and where it may be too low-yield to be worthwhile. Although the papers under review did not explicitly detail dual method use, other studies have demonstrated that women living with HIV are more likely to use dual methods after testing [31, 32].

In addition to HTS, there may be other services that could be efficiently and effectively integrated into FP for more comprehensive SRH service coverage. Perinatal transmission of HIV *and* syphilis remain significant causes of perinatal morbidity and mortality as both sexually transmitted infections can occur during pregnancy, delivery, or breastfeeding [33]. Current WHO recommendations include HIV and syphilis testing for all pregnant women at the first antenatal care visit [34]. While there is no WHO recommendation at this time regarding offering women syphilis screening and testing in FP services, offering both HIV testing and syphilis testing in FP services may further improve health outcomes for women and girls.

One fear of integration is that tasking providers with too many services may reduce the quality of these services. However, integration can yield positive effects on service quality as well as client outcomes for contraceptive use, antiretroviral therapy in pregnancy and HIV testing [32]. Recent evidence suggests that technical quality of client-provider consultations for the integration of HTS into FP services, as measured by both health facility structural and provider factors, showed improvement in Kenya [35]. Mayhew et al. also found that when health providers are supported by management, including a consistent supply of both HIV test kits and contraceptives, they feel motivated and welcome the teamwork and support from fellow providers – then integration is more likely to happen [36]. In Namibia, integrated HIV/SRH services improved accessibility, stigma, quality of antenatal care, and nurse productivity, while reducing time in the health facility without compromising uptake of care or services [37]. However, weak health system issues need to be addressed if integration is to work well. In one study, lower level facilities were more likely to offer HTS, but the same women were less likely to receive FP than at hospitals [29]. Qualitative data from interviews with health care providers delivering integrated services in Kenya were mixed at both the individual and operational levels. Although providers enjoyed improving their skill set and seeking improved client satisfaction, further work is required to explore what drives efficiency and interventions that may facilitate efficiency improvement of integration services [38, 39]. There is a need to resolve health systems obstacles to enable scale-up of integrated service provision [10, 19].

Limitations of this review include the fact that we may not have identified all eligible studies, despite conducting a systematic search and screening process. Findings of the review are also limited by the sparse existing evidence base. Our inclusion criteria focused on comparative designs measuring outcomes of interest either before and after an intervention or between intervention and comparison groups. However, for many of our outcomes, our included studies only presented non-comparative measures. For some outcomes, such as new cases of HIV and linkage to HIV care and treatment, it was clearly not possible for studies to provide comparative data for the period when HTS was not offered. For other outcomes, such as provider knowledge and attitudes about integrating HTS, it may have made sense to only ask these questions after the service integration has occurred. While we recognize these challenges within designs of the included studies, a lack of comparative outcomes prevents us from being able to make comparisons between integrated and non-integrated services.

## Conclusions

Global progress and success for reaching SRH and HIV targets is dependent on progress in sub-Saharan Africa where women and girls bear a high burden of both unintended pregnancy and sexually transmitted infections, including HIV. While significant attention has been paid to integrating family planning into HIV services, less attention has been given to using family planning services as a site for integration of HIV testing services. While there continues to be important progress, particularly in the highest burden countries of east and southern Africa, with greater attention to SRH/HIV integration, the time is right to encourage implementation of such service linkages in appropriate settings and evaluation of integrated services to strengthen the evidence base. In addition, there exist tools with which countries can monitor and assess impact on integrated service delivery on SRH/HIV linkages, including in particular the SRHR and HIV Linkages Index which combines 30 indicators to provide the first ever composite score towards achieving a linked response to SRHR and HIV [40]. Index scores and data are available for 60 countries, including for most countries in sub-Saharan Africa. Where integrated services are offered for FP and HTS, these must be based on respect and fulfillment of reproductive rights and should never be coerced. When availing of opportunities to strengthen such key integration efforts and offering counselling and services in a non-judgmental manner, with the full range of options and accurate information, this has the potential to increase the health and well-being of women and girls.

A French translation of this article has been included as Additional file 1.

A Portuguese translation of the abstract has been included as Additional file 2.

## Additional files

Additional file 1: Translation of this article into French. (PDF 304 kb)

Additional file 2: Translation of the abstract of this article into Portuguese. (PDF 97 kb)

## Abbreviations

FP: Family planning
HTS: HIV testing services
PICO: Population, intervention, control, outcome
PITC: Provider-initiated testing and counseling
SRH: Sexual and reproductive health
VHT: Village health teams
WHO: World Health Organization
