# Supplementary material for: Integration of HIV testing services into family planning services: a systematic review
Source: Reprod Health. 2019 May 29;16(Suppl 1):61. doi: 10.1186/s12978-019-0714-9 (PMC6538541; doi:10.1186/s12978-019-0714-9)
Supplement: Supplementary file 1 — Translation of this article into French. (PDF 304 kb) [file 12978_2019_714_MOESM1_ESM.pdf]

Reproductive Health 2019, 16 (Suppl 1) [translated from the original publication]

Intégration des services de dépistage du VIH dans les services de planning familial : un examen systématique

Manjulaa Narasimhan1*, Ping Teresa Yeh[2], Sabina Haberlen[3], Charlotte E. Warren[4], Caitlin E. Kennedy[2]
[1]Department of Reproductive Health and Research and UNDP/UNFPA/UNICEF/WHO/World Bank Special Programme, World Health Organization, Geneva, Switzerland
[2]Department of International Health, Johns Hopkins Bloomberg School of Public Health, Baltimore, Maryland, USA
[3]Department of Epidemiology, Johns Hopkins Bloomberg School of Public Health, Baltimore, Maryland, USA
[4]Population Council, Washington, District of Columbia, USA

*Auteur-ressource : Manjulaa Narasimhan: narasimhanm@who.int
Adresses électroniques des auteurs :
Ping Teresa Yeh: teresa.yeh@jhu.edu
Sabina Haberlen: shaberlen@jhu.edu
Charlotte E. Warren: cwarren@popcouncil.org
Caitlin E. Kennedy: caitlinkennedy@jhu.edu

Résumé

Contexte : En dépit d'un intérêt marqué pour l'intégration des services de santé sexuelle et reproductive (SSR) dans les services de lutte contre le VIH, la réciproque n'a pas fait l'objet d'autant d'intérêt. Lorsque les femmes et les jeunes filles courent un risque de contracter le VIH, proposer des services de dépistage du VIH lors de leurs visites aux services de planning familial (PF) offre d'importantes opportunités de répondre simultanément aux besoins liés au VIH et aux grossesses non désirées.

Méthodes : Nous avons procédé à une revue systématique des études comparant les services de PF avec SDV intégré à ceux sans SDV intégré ou avec un niveau d'intégration inférieur (par exemple, services d'orientation par rapport à des services sur site), sur les résultats suivants : adoption/conseil/offre de SDV, nouveaux cas de VIH identifiés, lien avec les soins et le traitement du VIH, utilisation de deux méthodes, satisfaction du client et qualité du service, et connaissances et attitudes des prestataires concernant l'intégration du SDV. Nous avons

effectué des recherches dans trois bases de données en ligne et inclus des études publiées dans une revue à comité de lecture avant la date de recherche du 20 juin 2017.

Résultats : Sur 530 citations identifiées, six études ont finalement répondu aux critères d'inclusion. Trois études ont été menées au Kenya et une en Ouganda, au Swaziland et aux États-Unis. La plupart se sont déroulées dans des cliniques de PF. Trois étaient organisées par l'Integra Initiative. La rigueur globale était modérée, avec un seul essai randomisé par grappes. L'adoption du SDV était généralement plus élevée sur les sites intégrés par rapport aux sites de comparaison ou de pré-intégration, notamment dans les analyses ajustées, même si les résultats variaient légèrement d'une étude à l'autre. Une étude a révélé que les femmes des sites intégrés étaient plus susceptibles d'être très satisfaites des services fournis, mais avaient des temps d'attente plus longs. Une étude a révélé une légère augmentation de la séropositivité au VIH chez les patients intégrés aux tests après intégration complète par rapport à un échantillon-test dédié. Aucune étude n'a analysé de manière comparative le lien entre les soins et le traitement du VIH, l'utilisation de deux méthodes ou les connaissances/attitudes des prestataires.

Conclusions : Les progrès et les succès mondiaux dans la réalisation des objectifs de SSR et de VIH dépendent des progrès accomplis en Afrique subsaharienne, où les femmes sont confrontées à de nombreuses grossesses non désirées et infections sexuellement transmissibles, y compris le VIH. Bien que la base de données probantes soit limitée, elle indique que l'intégration du SDV dans les services de PF est réalisable et offre un potentiel de résultats conjoints positifs. Le succès et l'extension de cette approche dépendront des besoins de la population et des facteurs du système de santé.

Mots clés : Intégration de services, Planning familial, VIH, Santé sexuelle et reproductive, Accès

Contexte

L'expansion à l'échelle mondiale du traitement antirétroviral a été le principal facteur à l'origine d'une baisse de 48 % du nombre de décès dus au SIDA, mais les maladies liées au SIDA restent une cause majeure de décès chez les femmes en âge de procréer (15-49 ans), en particulier en Afrique subsaharienne.[1] Lorsque les femmes et les jeunes filles courent un risque de contracter le VIH, proposer des services de dépistage du VIH lors de leurs visites aux services de planning familial (PF) offre d’importantes opportunités de répondre simultanément aux besoins liés au VIH et aux grossesses non désirées. La stratégie mondiale de l’Organisation mondiale de la santé (OMS) en matière de santé reproductive définit une approche globale de la santé sexuelle et reproductive (SSR) intégrant le VIH [2] et l’intégration des services de dépistage du VIH dans les services de PF peut contribuer à atteindre des objectifs communs en matière de santé et de droit humain, ainsi qu'à accélérer les progrès permettant un accès à des services de SSR complets et une amélioration des droits.[3, 4]

Une attention particulière a été accordée à l’intégration des services de PF aux services de dépistage, de prise en charge et de traitement du VIH[5-7] et à la fourniture de conseils de PF aux femmes vivant avec le VIH[8-10], mais peu d'intérêt a été porté à l’intégration du SDV dans les structures de PF, même s’il est prouvé que le service de dépistage facultatif du VIH intégré

dans les cliniques de PF peut potentiellement augmenter les taux d'adoption du test, de réception des résultats du test et de diagnostics de séropositivité chez les adolescents et les jeunes adultes.[11] Les patients du PF sexuellement actifs, notamment ceux qui vivent dans des environnements à forte prévalence du VIH ou qui adoptent des comportements qui les exposent à un risque plus élevé de contracter le VIH, peuvent également bénéficier du SDV. Une analyse systématique des liens multidirectionnels entre les services de planning familial et de lutte contre le VIH réalisée en 2009 a permis de recenser deux études qui fournissaient un SSD aux patients des centres de planning familial.[12] Une étude réalisée en République dominicaine a ajouté le SDV et le traitement du VIH aux services de PF fournis dans une clinique ;[13] une autre étude sud-africaine a comparé la prestation du SDV sur place aux patients des cliniques de PF ayant été orientés par une structure extérieure pour SDV.[14] Cependant, ni l'une ni l'autre n'ont été publiées en tant qu'articles revus par des pairs. Une autre étude approfondie réalisée en 2009 a analysé l'impact de l'intégration de tout élément de prévention, de soins et de traitement des IST ou du VIH dans les consultations de PF. Les données recueillies ont démontré le potentiel d'intégration de services tels que la satisfaction des patients et la réduction de la stigmatisation du VIH dans les cliniques. Par exemple, l'intégration des services de SSR et de VIH au Botswana a révélé une grande satisfaction de la part des patients (82,7 %), notamment parce qu'ils estimaient que l'intégration réduisait le nombre de visites au centre de santé.[15] Néanmoins, il était évident que les prestataires manquaient souvent l'occasion d'intégrer les soins ainsi que d'autres problèmes liés au programme pour maintenir la qualité des soins.[16] Hormis une étude Cochrane de 2012 portant sur l'intégration bidirectionnelle des services liés au VIH/sida dans les services de

santé maternelle, néonatale et infantile, de nutrition et de PF,[17] il n'y a pas eu d'examen systématique plus récent spécifique à l'intégration du SDV dans les services de PF, ce qui reste une lacune programmatique importante dans la prestation de ces services.

Ce document analyse les preuves de l'intégration du SDV dans les services de PF. Nous espérions identifier quels modèles d'intégration desdits services avaient été évalués, ainsi que leurs résultats positifs et négatifs.

Méthodes

Définitions

Pour les besoins de cette analyse, nous avons utilisé les définitions suivantes :

- Les **liens** font référence aux synergies bidirectionnelles dans les politiques, les systèmes et services entre la santé sexuelle et reproductive, les droits et le VIH. Ils font référence à une approche plus large fondée sur les droits de l'homme, dont l'intégration de services de SSR est un sous-ensemble.[18, 19]
- L'**intégration** fait référence au niveau de prestation de services et peut être comprise comme une adhésion à des programmes opérationnels pour garantir des résultats effectifs impliquant plusieurs modalités (telles que des fournisseurs à tâches multiples, des services d'orientation et des guichets uniques sous une même structure).[18]

L'OMS, le FNUAP, l'IPPF et ONUSIDA ont mis au point un cadre pour les liens SSR/VIH et ont défini l'intégration au niveau de la prestation de services comme « différents types de services ou programmes opérationnels de MNCHN [santé maternelle, néonatale et infantile, nutrition]

et VIH ensemble pour assurer et peut-être maximiser les résultats collectifs. »[20] Pour les besoins de cette analyse, nous utilisons cette définition d'intégration et nous nous concentrons sur le niveau de prestation de services, même si nous reconnaissons les nombreuses autres définitions existantes.

- L'OMS définit les **services de dépistage du VIH (SDV)** comme « l'ensemble des services devant être fournis avec le dépistage du VIH - conseil (informations préalables au test et conseil après le test) ; lien avec des services appropriés de prévention, de traitement et de soins du VIH et d'autres services cliniques et de soutien ; et la coordination avec les services de laboratoire pour optimiser l'assurance qualité et la fourniture de résultats corrects. »[21]

- Le **planning familial et la contraception** présentent des avantages directs pour la santé, tels que la prévention des grossesses non désirées et, par conséquent, la réduction de la mortalité et de la morbidité maternelles.[22] Les services de planning familial peuvent inclure diverses méthodes de contraception, mais aussi répondre aux besoins des individus en matière de fertilité, conseils et services de conseil en matière de grossesse, planification de la conception, services de base pour l'infertilité, services de santé préconception, dépistage et traitement des infections sexuellement transmissibles et services de planning familial postpartum.

Question PICO (Patient, Intervention, Comparaison, Outcome [résultat])

PICO : Le SDV devrait-il être intégré aux services de PF ?

P : Patients des services de PF

I : SDV intégré aux services de PF

C : Services de PF sans SDV intégré, ou avec un niveau d'intégration inférieur (par exemple, orientation vers une autre structure au lieu de services sur place)

O : (1) adoption, conseil ou offre de SDV, (2) nouveaux cas de VIH identifiés, (3) lien avec les soins et le traitement du VIH, (4) utilisation de deux méthodes, (5) satisfaction de la patientèle et qualité du service, (6) connaissances et attitudes des prestataires sur l'intégration de SDV

Processus de recherche et de sélection

Pour être inclus dans l'analyse, un article devait répondre aux critères d'inclusion suivants :

1) Étude comparative portant sur les utilisateurs de services de PF (avec ou sans partenaires) ayant reçu des services de PF sur des sites avec SDV intégré, par rapport aux patients de PF ayant reçu des services de PF sans SDV intégré, ou présentant un niveau d'intégration inférieur, sur un ou plusieurs des résultats clés décrits dans la question PICO ci-dessus.
2) Publié ou accepté pour publication dans une revue à comité de lecture avant la date de recherche du 20 juin 2017.

Les services de PF ont été considérés comme tout site où des services de PF sont systématiquement fournis, y compris des cliniques de PF indépendantes, des services de santé itinérants ou des sites fournissant des services de PF. Nous avons inclus les services de PF

postpartum ; toutefois, nous avons exclu les services de soins prénataux, car l'intégration du test de dépistage du VIH dans les soins prénataux a déjà été largement prise en compte dans le cadre des programmes de prévention de la transmission verticale.

Les études comparant les tests de dépistage du VIH acceptés ou facultatifs dans les services de PF n'ont pas été incluses, car ces études sont considérées comme deux approches différentes du test du VIH, plutôt que des modèles d'intégration différents. Il n'y avait aucune contrainte par langue ou zone géographique.

Trois bases de données électroniques ont été consultées jusqu'au 20 juin 2017 : PubMed, CINAHL (Cumulative Index to Nursing and Allied Health Literature [Index cumulatif de la littérature infirmière et relative à la santé]) et EMBASE. Les termes suivants ont été utilisés pour effectuer des recherches sur PubMed et adaptés au vocabulaire contrôlé des autres bases de données : (« test VIH* » [tiab] OU « conseil et dépistage du VIH » [tiab] OU « conseil et dépistage du VIH » [tiab]) ET (« services de planning familial » [mesh] OU contracepti* [tiab] OU « planning familial » [tiab] OU « planning familial postpartum » [tiab]).

Une recherche de référence secondaire a été effectuée sur toutes les études incluses dans l'analyse et une analyse précédente connexe.[12] Nous avons également contacté des auteurs d'études en cours sur ce sujet, notamment l'Integra Initiative[23], afin d'identifier d'autres articles.

Les titres, les résumés, les informations sur les citations et les termes descriptifs des citations identifiées grâce à la stratégie de recherche ont été initialement sélectionnés par un membre du personnel de l'étude. Les résumés restants ont été examinés en double exemplaire par deux examinateurs travaillant de manière indépendante, le règlement des éventuelles divergences ayant été confié à un membre expérimenté de l'équipe d'étude. Deux examinateurs indépendants ont évalué l'éligibilité des articles en texte intégral pour déterminer la sélection de textes pour l'étude finale.

Extraction et analyse des données

Les données ont été extraites à l'aide de formulaires standardisés. Les informations suivantes, adaptées de l'analyse précédente,[12] ont été recueillies à partir de chaque étude incluse : Référence de l'étude, pays, contexte (urbain/rural), paramètre (type de clinique/service), groupe cible, années de programme, années d'évaluation, nom du programme, intervention, format d'intégration (sur site, orientation, etc.), coût des services, plan d'étude, unité d'analyse, taille de l'échantillon, âge des patients de l'échantillon, sexe des patients de l'échantillon, durée du suivi, résultats et résultats rapportés.

La rigueur de l'étude a été évaluée à l'aide d'un outil composé de neuf éléments : (1) Plan de l'étude : données avant/après intervention, (2) Plan de l'étude : groupe témoin ou groupe de comparaison, (3) Plan de l'étude : cohorte, (4) Groupes de comparaison équivalents au départ sur le plan socio-démographique, (5) Groupes de comparaison équivalents au départ sur les

mesures de résultats, (6) Affectation aléatoire (groupe ou individu) à l'intervention, (7) Participants choisis au hasard pour évaluation, (8) Contrôle des facteurs de confusion potentiels, (9) Taux de suivi >= 75 %. Cette échelle est basée sur l'échelle d'évaluation de la rigueur en huit points précédemment développée pour les examens systématiques des interventions comportementales liées au VIH.[24, 25]

Les données ont été analysées de manière descriptive. En raison du manque d'études similaires avec des résultats combinables, la méta-analyse n'a pas été possible.

Résultats

Résultats de la recherche

La recherche initiale dans la base de données a donné 530 références, une référence étant identifiée par d'autres moyens ; il restait 374 références après la suppression des doublons (Figure 1). La sélection initiale a exclu 337 références et la sélection secondaire en a exclu 24 pour non-respect des critères d'inclusion. Après avoir examiné et discuté de manière approfondie les 13 articles restants, sept autres ont été exclus. Au final, six articles répondaient aux critères d'inclusion.[26-31] Le Tableau 1 décrit les paramètres, les méthodes et les résultats de l'étude. Le Tableau 2 présente une évaluation de la rigueur de l'étude.

Figure 1. Organigramme PRISMA montrant la disposition des résultats de recherche

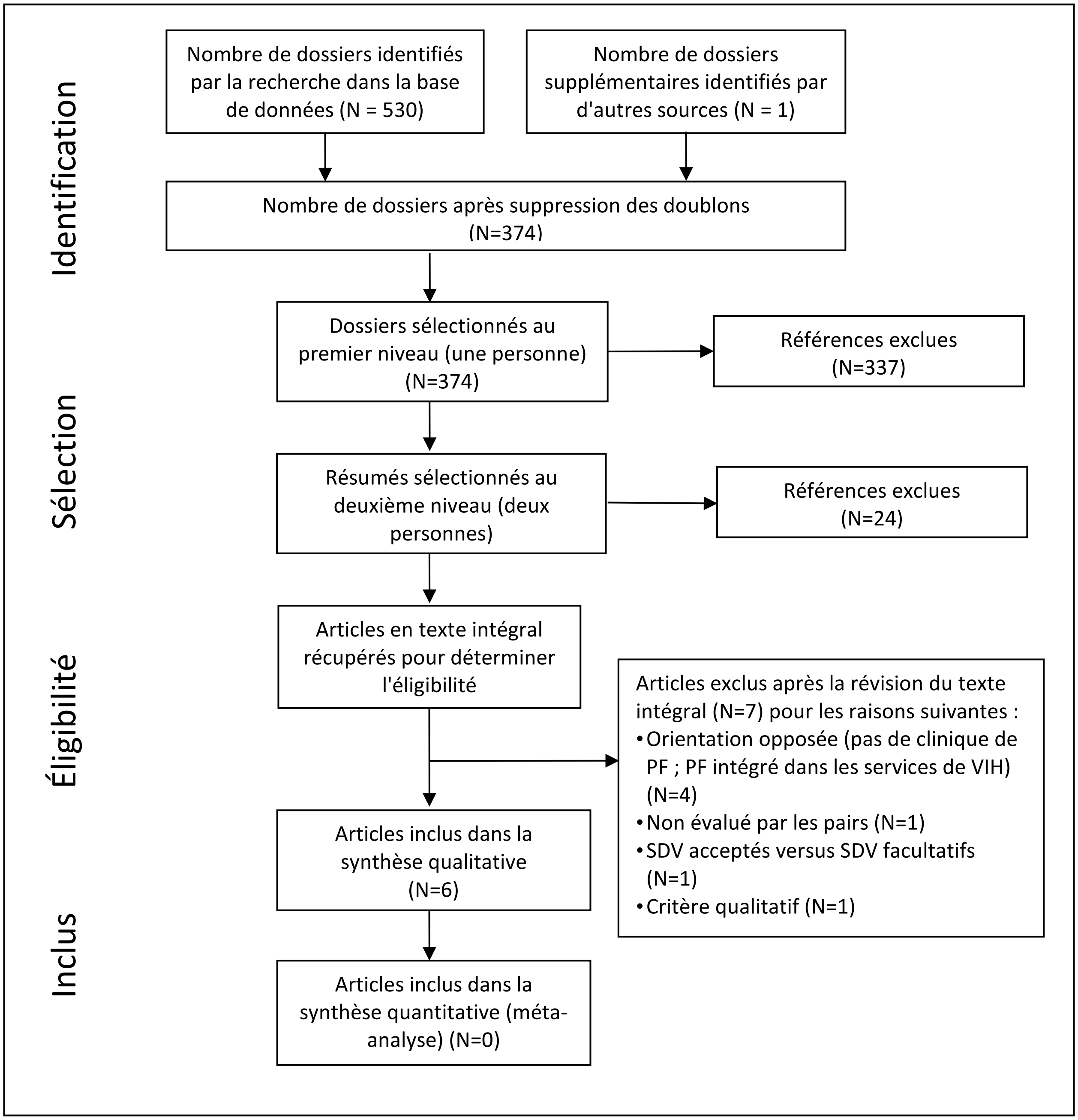

Descriptions des études

Cinq des six études provenaient d'Afrique subsaharienne (Tableau 1). Trois ont été menées au Kenya, [28, 30, 31] et une en Ouganda,[27] au Swaziland[26] et aux États-Unis d'Amérique.[29]

Trois ont été menées dans le cadre de l'Integra Initiative.[26, 28, 30] La plupart des études ont été menées dans des cliniques de PF, bien que deux soient en postnatal.[26, 30] La plupart des services ont été fournis dans des cliniques statiques, avec des tests sur site et des services de conseil et d'orientation vers les services de soins et de traitement du VIH destinés aux personnes dont les résultats du test du VIH sont redevenus positifs. Cependant, une étude a évalué l'efficacité des équipes de santé de village (ESV) formées pour proposer les SDV avec le service de PF ; les ESV dépendaient des centres de santé pour la supervision, l'approvisionnement en produits de base et la gestion des orientations des patients.[27] Toutes, sauf une, étaient centrées sur les besoins des patients.

La rigueur générale était modérée (Tableau 2). Il y a eu un essai randomisé en groupes et quatre essais non randomisés en groupes ; l'étude restante était une cohorte rétrospective. La plupart des études ont délibérément sélectionné des établissements, puis défini des échantillons de patients au sein de ces établissements. Trois études ont contrôlé les facteurs de confusion potentiels dans leurs analyses.

Résultats des études : Adoption/conseil/offre de SDV

L'adoption du SDV, mesurée de différentes manières, était le résultat le plus souvent observé dans les six études. L'adoption du SDV était généralement plus élevée sur les sites intégrés par rapport aux sites de comparaison ou de pré-intégration, notamment dans les analyses ajustées, même si les résultats variaient légèrement d'une étude à l'autre.

En Ouganda, l'essai randomisé par grappes évaluant les ESV a révélé que les participants du groupe d'intervention étaient beaucoup plus susceptibles que les participants du groupe témoin de déclarer avoir déjà fait un test de dépistage du VIH (99,27 % contre 94,96 %, p =

0,002) et avoir eu plus de tests de dépistage du VIH au cours des 12 derniers mois (p = 0,043).[27]

Au Kenya, le planning familial intégré et le conseil et le dépistage proposés par le prestataire (CDPP) dans les hôpitaux, les centres de santé et les dispensaires du secteur public ont été associés à des pourcentages nettement plus élevés de test de dépistage du VIH pour les nouveaux patients (74 % contre 34 %) et nouvelles visites de patients existants (56 % contre 27 %), par rapport à un modèle de référence.[31] Même si la proportion de nouveaux patients et de patients existants ayant refusé le test quand on leur a proposé de le faire n'a pas été significativement plus élevée, la proportion de patients dépistés a été considérablement plus élevée (35 % contre 20 %), pour les nouveaux patients (37 % contre 22 %) comme pour les patients existants revenant faire une visite (34 % contre 19 %).

Aux États-Unis, une clinique de PF urbaine financée par le Titre X est passée du recours à un conseiller désigné en matière de VIH pour un SDV ciblé à un modèle utilisant le personnel de la clinique pour fournir un SDV, régulier, non ciblé et rapide comme norme de soins dans le centre de PF (intégration complète).[29] Les taux d'acceptation des tests sont passés de 76 % au cours de la période désignée du testeur du VIH à 89 % en intégration complète ; de même, le pourcentage de patients avec un test VIH documenté dans leur dossier médical au cours des 12 derniers mois est passé de 34 % avant l'intégration d'un SDV à 65 % pendant la période d'échantillon-test du VIH désignée à 71 % en intégration complète.

Deux études, toutes deux réalisées par l'Integra Initiative, ont examiné les structures de soins postnataux. Au Kenya, où les services intégrés du VIH et de PF dans les soins postnataux ont été comparés aux services indépendants, les probabilités d’adoption du CDPP étaient plus élevées

dans les sites d’intervention que dans les sites de comparaison (aOR=1,6, p<0,01, IC 95 % : 1,2-2,2).[30] Au Swaziland, les activités et les ressources visant à renforcer l'intégration des services liés au VIH dans les services de soins postnataux comprenaient un programme de formation visant à faciliter l'encadrement des prestataires de soins de santé de première ligne, des outils de travail pour promouvoir l'intégration et un soutien continu pour clarifier les rôles, discuter des changements organisationnels, des orientations/liens et de la gestion des statistiques de service.[26] Les services de conseil sur le VIH ont augmenté dans deux sites d’intervention et deux sites de comparaison et ont diminué dans un site d’intervention et deux sites de comparaison ; un établissement d'intervention n'a pas montré de changement significatif sur ce point. Toutefois, l'étude a également indiqué qu'il était impossible de préciser quels sites intégraient réellement quels services.

Dans l’étude finale menée par l'Integra Initiative au Kenya, l’intégration SSR/VIH a ajouté les services suivants à la prestation de services standard du PF : discussion sur les désirs de fertilité, encouragement à l'utilisation/fourniture de préservatifs, évaluation du risque d'IST/de VIH, contrôle du VIH, offre de SDV, dépistage du cancer du col de l'utérus, services de traitement pré-VIH et/ou orientation vers une unité de traitement du VIH pour les patients séropositifs.[28] La proportion de patients ayant déclaré avoir effectué un test de dépistage du VIH depuis le dernier entretien est passée de 8,4 % au début de l'étude à 71,8 % au moment de la visite de suivi de 24 mois dans le groupe d'intervention, contre 47,6 % à 60,7 % pour le groupe témoin. Le pourcentage de femmes atteignant ce que l'étude considérait comme des objectifs de dépistage du VIH (minimum de deux tests, un test par an) au cours de la cohorte de deux ans, était en réalité plus élevé dans le groupe de comparaison que dans le groupe

d'intervention (p<0,05). Cependant, parmi les patients ayant reçu des services intégrés au départ, quel que soit le groupe (71 %), par rapport à ceux qui n'en ont pas reçu (61 %) (p<0,01). De plus, les femmes présentant l'exposition cumulée la plus élevée aux services intégrés étaient plus susceptibles d'avoir satisfait à l'exigence de test (77 %) par rapport au groupe à score moyen (71 %) et au groupe à score faible (60 %) (p<0,001).

Résultats des études : Nouveaux cas de VIH et lien avec les soins et le traitement du VIH

Aucune étude n'a analysé de manière comparative les nouveaux cas de VIH identifiés (résultat) ou le lien avec les soins et le traitement du VIH. Cependant, une étude américaine a mesuré le taux de séropositivité (sans préciser davantage s'il s'agissait de cas nouveaux ou déjà diagnostiqués).[29] Alors que les taux de séropositivité n'étaient pas disponibles pour la période précédant les tests rapides, <0,5 % de l'ensemble des patients étaient dépistés séropositifs pendant la période d'échantillon-test désigné, tandis que 0,7 % des patients (0,6 % des femmes et 10 % des hommes) étaient dépistés séropositifs pendant la période d'intégration complète. Bien qu'aucune donnée comparative n'ait été présentée pour établir un lien entre les soins et le traitement du VIH, deux études ont noté le nombre total de patients testés positifs (de 3 à 16 personnes) et ont noté qu'ils étaient tous liés aux soins médicaux.[27, 29]

Résultats des études : Utilisation de double méthode

Aucune étude n'a fourni de données comparatives sur l'utilisation de la méthode double.

Résultats des études : Satisfaction de la patientèle et qualité du service

Une seule étude a rapporté des indicateurs comparatifs (pré-post ou multi-bras) sur la satisfaction du patient et les perceptions de la qualité de service autour de l'intégration du SDV dans les services de PF. Cette étude de l'Integra Initiative au Kenya a utilisé un score moyen basé sur les échelles de Likert sur « l'évaluation globale du service, les coûts, le temps d'attente, la disponibilité des médicaments et des fournitures, la possibilité de recevoir d'autres services simultanément, les heures d'ouverture, la convivialité des prestataires, la disponibilité des médecins/infirmiers(ères), l'écoute des prestataires, la possibilité pour le patient de poser des questions. »[28] Les femmes des sites d'intervention étaient plus susceptibles d'être très satisfaites des services (30 % contre 27 %), mais attendaient plus de 30 minutes (57 % contre 0,2 %) et étaient moins susceptibles d'avoir déboursé des frais (83 % contre 93 %).

L'essai randomisé par grappes d'ESV en Ouganda comprenait des mesures non comparatives de la satisfaction de la patientèle et de la qualité du service dans le groupe d'intervention uniquement.[27] Plus de 95 % des patients testés par une ESV ont répondu de manière positive aux questions concernant leur satisfaction sur les relations interpersonnelles et les informations et services reçus. La grande majorité (99,1 %) a également déclaré avoir confié des informations confidentielles à l'ESV. Tous les clients testés par l'ESV et séronégatifs au VIH avaient l'intention de se faire à nouveau dépister à l'avenir, et 93,5 % ont déclaré préférer faire appel à une ESV pour leur prochain dépistage. Le score de connaissances combiné moyen des ESV était de 5,1 sur 7 points possibles, avec 81,6 % des ESV ayant obtenu au moins 5 ; les principales lacunes en matière de connaissances étaient la fréquence recommandée pour la répétition des tests parmi les patients séronégatifs au VIH et les mesures de sécurité. Parmi les 34 ESV ayant participé au

contrôle d'assurance qualité, 85,3 % ont réussi avec un score de 100 % de concordance avec le laboratoire de référence. Les informations rapportées par les patients suggèrent qu'aucun patient testé par une ESV n'a signalé de problèmes avec les procédures de prélèvement par piqûre aux doigts. La majorité des patients ont déclaré que les ESV leur avaient transmis des informations/conseils clés sur le SDV.

Résultats des études : Connaissances et attitudes des prestataires sur l'intégration du SDV

Aucun résultat comparatif n'a été présenté sur les connaissances et les attitudes des prestataires concernant l'intégration du SDV. Cependant, une étude américaine a évalué l'attitude des prestataires six mois après l'intervention avec un taux de réponse de 70 %.[29] En utilisant une échelle de Likert, 100 % des répondants ont jugé « très important » de proposer le dépistage systématique du VIH à tous les patients, 78 % ont jugé « très » ou « plutôt réussie » l'intégration du test de dépistage du VIH et 56 % ont déclaré avoir effectué un test de dépistage du VIH en clinique. Tous les membres du personnel ont estimé que le soutien sur site de conseillers en VIH expérimentés était « le plus utile ».

Discussion

Bien que la base de données probantes soit limitée, les études existantes indiquent que l'intégration du SDV dans les services de PF est réalisable et offre un potentiel de résultats positifs. Les six articles décrits ici évaluaient le taux d'adoption du SDV comme résultat principal. Cependant, les cinq autres éléments mesures de résultats que nous avons sélectionnés *a priori* (nouveaux cas de VIH identifiés, lien avec les soins et le traitement du VIH,

utilisation de deux méthodes, satisfaction du client et qualité du service, connaissances et attitude des prestataires concernant l'intégration du SDV) offraient une capacité de mesure comparative limitée.

Il est fâcheux que dans la littérature existante, aucune étude ne fournisse de mesures comparatives des nouveaux cas de VIH identifiés et d'utilisation de méthodes doubles, et que peu d'entre elles fournissent des mesures comparatives des liens avec les soins et les traitements, la satisfaction de la patientèle et la qualité du service, ou les connaissances et attitudes des prestataires concernant l'intégration du SDV. Le test de dépistage du VIH a pour objectif d'identifier les personnes vivant avec le VIH qui n'ont pas encore reçu de diagnostic. L'étape suivante consiste à les orienter vers les services de lutte contre le VIH ; et pour celles qui ont un test séronégatif au VIH, d'avoir accès aux interventions de prévention afin qu'elles ne soient pas contaminées par le virus. Par conséquent, il est essentiel de disposer d'informations sur le nombre de cas et de renforcer les liens avec les soins, notamment pour identifier les domaines dans lesquels l'intégration du SDV dans les services de PF est judicieuse et où le rendement peut être trop faible pour en valoir la peine. Bien que les documents examinés ne détaillent pas explicitement l'utilisation de la double méthode, d'autres études ont montré que les femmes vivant avec le VIH étaient plus susceptibles d'utiliser une double méthode après le test.[32, 33]

Outre le SDV, il est possible que d'autres services puissent être intégrés de manière efficace dans le PF pour une couverture plus complète des services de SSR. La transmission périnatale

du VIH et de la syphilis demeure une des causes importantes de morbidité et de mortalité périnatales, car les deux infections sexuellement transmissibles peuvent survenir pendant la grossesse, l'accouchement ou l'allaitement.[34] Les recommandations actuelles de l'OMS incluent le dépistage du VIH et de la syphilis chez toutes les femmes enceintes lors de la première visite de soins prénataux.[35] Bien que l'OMS ne fasse actuellement aucune recommandation concernant le dépistage et les tests de syphilis dans les services de PF, proposer des tests de dépistage du VIH et de dépistage de la syphilis dans les services de PF pourrait encore améliorer les résultats pour la santé des femmes et des jeunes filles.

L'une des craintes de l'intégration est que le fait de charger des fournisseurs avec trop de services peut en réduire la qualité. Cependant, l'intégration peut avoir des effets positifs sur la qualité du service ainsi que sur les résultats obtenus par les patients notamment pour l'utilisation de la contraception, le traitement antirétroviral pendant la grossesse et le dépistage du VIH.[33] Des données récentes suggèrent que la qualité technique des consultations patient-prestataire pour l'intégration du SDV dans les services de PF, mesurée à la fois par les facteurs structurels des prestataires de santé et par les prestataires, s'est améliorée au Kenya.[36] Mayhew et al. ont également constaté que lorsque les prestataires de santé sont soutenus par la direction, notamment avec un approvisionnement constant en kits de dépistage du VIH et en contraceptifs, ils se sentent motivés et se félicitent du travail d'équipe et du soutien d'autres prestataires : l'intégration est alors plus susceptible de se produire.[37] En Namibie, les services intégrés de VIH/SSR ont permis de réduire la stigmatisation et d'améliorer l'accessibilité, la qualité des soins prénataux et la productivité du personnel infirmier, tout en réduisant le temps

passé dans l'établissement de santé sans compromettre la prise en charge des soins ou les services.[38] Toutefois, pour que l'intégration fonctionne correctement, il est nécessaire de remédier aux problèmes des systèmes de santé fragiles. Dans une étude, les établissements de taille inférieure étaient plus susceptibles de proposer un SDV, mais les mêmes femmes étaient moins susceptibles d'accéder aux services de PF que dans les hôpitaux.[30] Les données qualitatives tirées d'entretiens avec des prestataires de soins de santé fournissant des services intégrés au Kenya étaient hétérogènes aux niveaux individuel et opérationnel. Bien que les prestataires aient apprécié d'améliorer leurs compétences et de rechercher une satisfaction supérieure de la patientèle, des efforts supplémentaires sont nécessaires pour explorer les facteurs d'efficacité et les interventions susceptibles de faciliter l'amélioration de l'efficacité des services d'intégration.[39, 40] Il est nécessaire de supprimer les contraintes rencontrées par les systèmes de santé pour optimiser les prestations de services intégrés.[10, 19]

Cette analyse est limitée par le fait que nous n'avons probablement pas identifié toutes les études éligibles, en dépit du processus de recherche et de sélection systématique. Les conclusions de l'analyse sont également limitées par la faible base de données probantes existante. Nos critères d'inclusion se sont concentrés sur les plans comparatifs mesurant les résultats d'intérêt, avant et après une intervention, ou entre les groupes d'intervention et de comparaison. Cependant, pour nombre de nos résultats, nos études incluses ne présentaient que des critères de mesure non comparatifs. Pour certains résultats, tels que les nouveaux cas de VIH et le lien avec les soins et le traitement du VIH, il n'était pas possible pour les études de fournir des données comparatives pour la période où le SDV n'était pas proposé. Pour d'autres

résultats, tels que les connaissances et les attitudes des prestataires de soins concernant l'intégration de SDV, il aurait peut-être été judicieux de poser ces questions uniquement après l'intégration du service. Bien que nous reconnaissions ces difficultés dans les plans des études incluses, le manque de résultats comparatifs nous empêche de pouvoir comparer les services intégrés et non intégrés.

Conclusions

Les progrès et les succès mondiaux dans la réalisation des objectifs de SSR et de VIH dépendent des progrès accomplis en Afrique subsaharienne, où les femmes et les jeunes filles sont confrontées à de nombreuses grossesses non désirées et infections sexuellement transmissibles, y compris le VIH. Une grande attention a été accordée à l'intégration du planning familial dans les services de lutte contre le VIH, mais une attention moindre a été accordée à l'éventuelle utilisation des services du planning familial comme structure d'intégration des services de dépistage du VIH. Bien que des progrès importants continuent d'être réalisés, en particulier dans les pays les plus touchés d'Afrique de l'Est et du Sud, en portant notamment une attention supérieure à l'intégration des services de SSR/VIH, il est temps d'encourager la mise en place de tels liens de services dans des contextes appropriés et l'évaluation de services intégrés pour renforcer la base de données probantes à ce sujet. En outre, il existe des outils permettant aux pays de surveiller et d'évaluer l'impact de la prestation de services intégrés sur les liens SSR/VIH, notamment l'Indice des liens SDSR/VIH, qui associe 30 indicateurs afin de fournir le tout premier score combiné permettant d'obtenir une réponse aux SDSR et au VIH.[41] Les scores et les données de l'indice sont disponibles pour 60 pays, y

compris pour la plupart des pays d'Afrique subsaharienne. Lorsque des services intégrés sont proposés pour le PF et le SDV, ceux-ci doivent être basés sur le respect et la réalisation des droits en matière de procréation et ne doivent jamais être contraints. S'ils saisissent des occasions de renforcer ces efforts d'intégration clés et offrent des conseils et des services sans porter de jugement, avec toute la gamme d'options et des informations précises, cela peut potentiellement améliorer la santé et le bien-être des femmes et des jeunes filles.

Liste des abréviations

PF : planning familial

OMS : Organisation mondiale de la Santé

SSR : santé sexuelle et reproductive

SDV : services de dépistage du VIH

PICO : population, intervention, comparaison, outcome (résultat)

ESV : équipes de santé de village

CDPP : conseil et dépistage proposés par le prestataire

Concernant ce supplément

Cet article a été publié comme partie de l'ouvrage *Reproductive Health*, Volume 16 Supplement 1, 2019: Effective Integration of Sexual Reproductive Health and HIV Prevention, Treatment, and Care Services across sub-Saharan Africa: Where is the evidence for program implementation?
Le supplément a été publié dans le cadre d'une collaboration entre *Reproductive Health* et *BMC Public Health.* L'intégralité du contenu, avec les versions en français, en portugais et en anglais, est disponible en ligne :
https://bmcpublichealth.biomedcentral.com/articles/supplements/volume-19-supplement-1
et
https://reproductive-health-journal.biomedcentral.com/articles/supplements/volume-16-supplement-1

Déclarations

Approbation éthique et accord de participation

Non applicable

Accord de publication

Non applicable

Disponibilité des données et matériels

Le partage de données n'est pas applicable à cet article car aucun ensemble de données n'a été généré ou analysé au cours de la présente étude.

Intérêts concurrents

Les auteurs déclarent ne pas avoir de conflits d'intérêts.

Financement

Ces travaux ont été financés par le Département Santé et recherche génésiques de l'OMS, notamment le Programme spécial de recherche, de développement et de formation à la recherche en reproduction humaine (HRP) et l'USAID (Agence des États-Unis pour le développement international). Les entités de financement ont participé à la planification de l'étude et à la rédaction du manuscrit.

Le supplément de la revue est rendu possible grâce au soutien généreux du peuple américain via la United States Agency for International Development (USAID) en partenariat avec le Fonds des Nations unies pour la population (FNUAP) et le Programme commun des Nations Unies sur le VIH/SIDA (ONUSIDA).

Les opinions exprimées dans la présente publication sont celles des auteurs et ne reflètent pas nécessairement les politiques officielles de l'USAID, du FNUAP ou de l'ONUSIDA, la mention des

dénominations de ministères ou d'organismes n'implique pas non plus l'aval du gouvernement américain, du FNUAP ou de l'ONUSIDA.

Contributions des auteurs

MN et CEK ont conçu le protocole d'étude. CEK a effectué les recherches dans les bases de données. CEK et PTY ont sélectionné et analysé les résultats de la recherche. PTY et SH ont extrait les données et synthétisé les résultats avec CEK. Tous les auteurs ont contribué à la finalisation du manuscrit. Tous les auteurs ont lu et approuvé le manuscrit final.

Remerciements

Un grand merci à James Kiarie (OMS/SRG) et à Rachel Baggaley (OMS/VIH) pour avoir examiné le protocole et fourni des commentaires oraux et écrits suite à leur analyse.

Références

1. UNAIDS: UNAIDS Reference Data 2017. Geneva, Switzerland: UNAIDS; 2017. Available from: http://www.unaids.org/sites/default/files/media_asset/20170720_Data_book_2017_en.pdf- Accessed 14 December 2018.
2. WHO: Reproductive health strategy to accelerate progress towards the attainment of international development goals and targets. Geneva: World Health Organization; 2004. Available from: http://apps.who.int/iris/bitstream/handle/10665/68754/WHO_RHR_04.8.pdf?sequence=1- Accessed 14 December 2018.
3. Askew I, Berer M. The contribution of sexual and reproductive health services to the fight against HIV/AIDS: a review. Reprod Health Matters. 2003; 11(22):51-73.
4. Kennedy CE, Spaulding AB, Brickley DB, Almers L, Mirjahangir J, Packel L, Kennedy GE, Mbizvo M, Collins L, Osborne K. Linking sexual and reproductive health and HIV interventions: a systematic review. J Int AIDS Soc. 2010; 13:26.
5. Haberlen SA, Narasimhan M, Beres LK, Kennedy CE. Integration of Family Planning Services into HIV Care and Treatment Services: A Systematic Review. Stud Fam Plann. 2017; 48(2):153-177.
6. Grossman D, Onono M, Newmann SJ, Blat C, Bukusi EA, Shade SB, Steinfeld RL, Cohen CR. Integration of family planning services into HIV care and treatment in Kenya: a cluster-randomized trial. AIDS. 2013; 27(Suppl 1):S77-85.
7. Cohen CR, Grossman D, Onono M, Blat C, Newmann SJ, Burger RL, Shade SB, Bett N, Bukusi EA. Integration of family planning services into HIV care clinics: Results one year after a cluster randomized controlled trial in Kenya. PLoS One. 2017; 12(3):e0172992.
8. O'Reilly KR, Kennedy CE, Fonner VA, Sweat MD. Family planning counseling for women living with HIV: a systematic review of the evidence of effectiveness on contraceptive uptake and pregnancy incidence, 1990 to 2011. BMC Public Health. 2013; 13:935.
9. Wilcher R, Cates W, Jr., Gregson S. Family planning and HIV: Strange bedfellows no longer. AIDS. 2009; 23(Suppl 1):S1-6.
10. Wilcher R, Hoke T, Adamchak SE, Cates W, Jr. Integration of family planning into HIV services: a synthesis of recent evidence. AIDS. 2013; 27(Suppl 1):S65-75.

11. Buzi RS, Madanay FL, Smith PB. Integrating Routine HIV Testing into Family Planning Clinics That Treat Adolescents and Young Adults. Public Health Rep. 2016; 131 (Suppl 1):130-138.
12. Spaulding AB, Brickley DB, Kennedy C, Almers L, Packel L, Mirjahangir J, Kennedy G, Collins L, Osborne K, Mbizvo M. Linking family planning with HIV/AIDS interventions: a systematic review of the evidence. AIDS. 2009; 23(Suppl 1):S79-88.
13. IPPF-WHR: Integrating HIV/AIDS treatment and care services into a family planning setting. Vol. 2017. London: IPPF; 2006. Available from: http://srhhivlinkages.org/wp-content/uploads/2013/04/spotlightintegratinghivfp_2006_en.pdf. Accessed 14 December 2018.
14. Mullick S, Khoza D, Askew I, Maluka T, Menziwa M: Integrating counseling and testing into family planning services: what happens to the existing quality of family planning when HIV services are integrated in South Africa? Linking reproductive health, family planning, and HIV/AIDS in Africa. Addis Ababa; 9-10 October 2006.
15. UNFPA, Botswana MOH, UNAIDS: Evaulation of the SRH/HIV Linkages Project in Botswana. Gabarone, Botswana: UNFPA Botswana; 2016. Available from: http://botswana.unfpa.org/en/publications/evaluation-srhhiv-linkages-project-botswana. Accessed 14 December 2018.
16. Church K, Mayhew SH. Integration of STI and HIV prevention, care, and treatment into family planning services: a review of the literature. Stud Fam Plann. 2009; 40(3):171-186.
17. Lindegren ML, Kennedy CE, Bain-Brickley D, Azman H, Creanga AA, Butler LM, Spaulding AB, Horvath T, Kennedy GE. Integration of HIV/AIDS services with maternal, neonatal and child health, nutrition, and family planning services. The Cochrane Database Syst Rev. 2012(9):CD010119.
18. Inter-Agency Working Group on SRH and HIV Linkages: SRHR and HIV Linkages: Navigating the work in progress 2017. Geneva: UNFPA, WHO, and IPPF; 2017. Available from: http://index.srhhivlinkages.org/docs/IAWG_navigating-the-work-in-progress-2017.pdf. Accessed 14 December 2018.
19. Warren CE, Hopkins J, Narasimhan M, Collins L, Askew I, Mayhew SH. Health systems and the SDGs: Lessons from a joint HIV and sexual and reproductive health and rights response. Health Policy Plan. 2017; 32(Suppl 4):iv102-iv107.
20. WHO, UNFPA, IPPF, UNAIDS: Sexual and reproductive health and HIV/AIDS: A framework for priority linkages. 2005. Available from: https://www.who.int/reproductivehealth/publications/linkages/HIV_05_5/en/. Accessed 14 December 2018.
21. WHO: Consolidated guidelines on HIV testing services. Geneva: World Health Organization; 2015. Available from: http://www.who.int/hiv/pub/guidelines/hiv-testing-services/en/. Accessed 14 December 2018.
22. Rodríguez MI, Say L, Temmerman M. Family planning versus contraception: what's in a name? Lancet Glob Health. 2014; 2(3):e131-e132.
23. Warren CE, Mayhew SH, Vassall A, Kimani JK, Church K, Obure CD, du-Preez NF, Abuya T, Mutemwa R, Colombini M *et al*. Study protocol for the Integra Initiative to assess the benefits and costs of integrating sexual and reproductive health and HIV services in Kenya and Swaziland. BMC Public Health. 2012; 12:973.
24. Denison JA, O'Reilly KR, Schmid GP, Kennedy CE, Sweat MD. HIV voluntary counseling and testing and behavioral risk reduction in developing countries: a meta-analysis, 1990-2005. AIDS Behav. 2008; 12(3):363-373.
25. Kennedy C, O'Reilly K, Medley A, Sweat M. The impact of HIV treatment on risk behaviour in developing countries: A systematic review. AIDS Care. 2007; 19(6):707-720.
26. Birdthistle IJ, Mayhew SH, Kikuvi J, Zhou W, Church K, Warren CE, Nkambule R, Fenty J. Integration of HIV and maternal healthcare in a high HIV-prevalence setting: Analysis of client flow data over time in Swaziland. BMJ Open. 2014; 4(3):e003715.
27. Brunie A, Wamala-Mucheri P, Akol A, Mercer S, Chen M. Expanding HIV testing and counselling into communities: Feasibility, acceptability, and effects of an integrated family planning/HTC service delivery model by Village Health Teams in Uganda. Health Policy Plan. 2016; 31(8):1050-1057.
28. Church K, Warren CE, Birdthistle I, Ploubidis GB, Tomlin K, Zhou W, Kimani J, Abuya T, Ndwiga C, Sweeney S *et al*. Impact of Integrated Services on HIV Testing: A Nonrandomized Trial among Kenyan Family Planning Clients. Stud Fam Plann. 2017; 48(2):201-218.
29. Criniti SM, Aaron E, Hilley A, Wolf S. Integration of routine rapid HIV screening in an urban family planning clinic. J Midwifery Womens Health. 2011; 56(4):395-399.
30. Kimani J, Warren CE, Abuya T, Ndwiga C, Mayhew S, Vassall A, Mutemwa R, Askew I. Use of HIV counseling and testing and family planning services among postpartum women in Kenya: A multicentre, non-randomised trial. BMC Womens Health. 2015; 15:104.
31. Liambila W, Askew I, Mwangi J, Ayisi R, Kibaru J, Mullick S. Feasibility and effectiveness of integrating provider-initiated testing and counselling within family planning services in Kenya. AIDS. 2009; 23(Suppl 1):S115-S121.
32. Kimani J, Warren CE, Abuya T, Mutemwa R, Integra Initiative, Mayhew S, Askew I. Family Planning use and fertility desires among women living with HIV in Kenya. BMC Public Health. 2015; 15:909.

33. Warren CE, Abuya T, Askew I, Integra Initiative. Family planning practices and pregnancy intentions among HIV positive and HIV negative postpartum women in Swaziland: A cross sectional survey. BMC Pregnancy Childbirth. 2013; 13:150.
34. Mabey D, Peeling RW. Syphilis, still a major cause of infant mortality. Lancet Infect Dis. 2011; 11(9):654-655.
35. WHO: Global Guidance on Criteria and Processes for Validation: Elimination of Mother-to-Child Transmission of HIV and Syphilis, 2nd ed. Geneva: World Health Organization; 2014. Available from: https://www.who.int/reproductivehealth/publications/emtct-hiv-syphilis/en/. Accessed 14 December 2018.
36. Mutemwa R, Mayhew SH, Warren CE, Abuya T, Ndwiga C, Kivunaga J. Does service integration improve technical quality of care in low-resource settings? An evaluation of a model integrating HIV care into family planning services in Kenya. Health Policy Plan. 2017; 32(Suppl 4):iv91-iv101.
37. Mayhew S. Numbers, systems, people: how interactions influence integration. Insights from case studies of HIV and reproductive health services delivery in Kenya. Health Policy Plan. 2017; 32(Suppl 4):iv67-iv81.
38. Zapata T, Forster N, Campuzano P, Kambapani R, Brahmbhatt H, Hidinua G, Turay M, Ikandi SK, Kabongo L, Zariro F. How to Integrate HIV and Sexual and Reproductive Health Services in Namibia, the Epako Clinic Case Study. Int J Integr Care. 2017; 17(4):1.
39. Obure CD, Sweeney S, Darsamo V, Michaels-Igbokwe C, Guinness L, Terris-Prestholt F, Muketo E, Nhlabatsi Z, Integra I, Warren CE *et al*. The Costs of Delivering Integrated HIV and Sexual Reproductive Health Services in Limited Resource Settings. PLoS One. 2015; 10(5):e0124476.
40. Mutemwa R, Mayhew S, Colombini M, Busza J, Kivunaga J, Ndwiga C. Experiences of health care providers with integrated HIV and reproductive health services in Kenya: a qualitative study. BMC Health Serv Res. 2013; 13(1):18.
41. WHO: The SRHR and HIV Linkages Index. Geneva, Switzerland: WHO/HRP; 2017. Available from: http://www.who.int/reproductivehealth/topics/linkages/indicators/en/. Accessed 14 December 2018.

Tableaux

Tableau 1. Descriptions des études incluses

Étude	Configuration	Intervention	Conception de l'étude	Taille de l'échantillon
Birdthistle et al., 2014 [24]	**Lieu :** Swaziland/unités urbaines, rurales et périurbaines/SMI (Services de santé maternelle et infantile) des établissements de santé du secteur public (gouvernement)<br><br>**Groupe cible :** Patients de sexe féminin	**Années de programme :** 2009-2012<br>**Années d'évaluation :** 2009-2012<br>**Nom :** Integra Initiative<br>Intervention : Activités et ressources pour renforcer l'intégration des services liés au VIH dans les soins postnataux :<br>(1) Programme de formation pour faciliter l'encadrement des prestataires de soins de santé de première ligne<br>(2) Outils de travail pour promouvoir l'intégration<br>(3) Support continu pour clarifier les rôles, discuter des changements organisationnels, des orientations/liens et de la gestion des statistiques de service<br>**Format :** Orientations sur place	**Plan de l'étude :** Essai non randomisé en groupes<br><br>**Sélection des sites :** Spécifique<br><br>**Sélection des participants** Consécutive	**Taille d'échantillon :** 3261 femmes ont été suivies en 2009,<br>2086 en 2010 et<br>2916 en 2012<br><br>**Âge :** N/A<br>**Sexe :** Féminin<br>**Suivi :** N/A
Brunie et al., 2016 [25]	**Lieu :** Ouganda / NR / centre de santé<br><br>**Groupe cible :**	**Années de programme :** 2012-2013<br>**Années d'évaluation :** 2013<br>**Nom :** N/A<br>**Intervention :** Équipes de santé villageoises formées pour proposer	**Plan de l'étude :** Essai randomisé en groupes<br><br>**Sélection des**	**Taille d'échantillon :** 256 patients<br><br>**Âge :** Moyenne (ET) : groupe d'intervention 31,02 (0,40),

Étude	Configuration	Intervention	Conception de l'étude	Taille de l'échantillon
	Patients	des SDV ainsi que des services de planning familial et dépendantes des centres de santé pour la supervision, la fourniture de produits de base et la gestion des orientations des patients<br>**Format :** Test sur place, orientation vers une clinique de santé	**sites :**<br>Spécifique<br><br>**Sélection des participants**<br>Systématique (tous les x patients)	groupe témoin 30,73 (1,14)<br>**Sexe :** N/A<br>**Suivi :** N/A
Church et al., 2017 [26]	**Lieu :**<br>Kenya/centres urbains, ruraux et périurbains/centres de santé et hôpitaux<br><br>**Groupe cible :**<br>Patientes du planning familial	**Années de programme :** 2009-2012<br>**Années d'évaluation :** 2010-2012<br>**Nom :** Integra Initiative<br>**Intervention :** L'intégration SSR/VIH a ajouté les services suivants à la prestation de services standard du PF : discussion sur les désirs de fertilité, encouragement à l'utilisation/fourniture de préservatifs, évaluation du risque d'IST/de VIH, contrôle du VIH, proposition de SDV, dépistage du cancer du col de l'utérus, services de traitement pré-VIH et/ou orientation vers une unité de traitement du VIH pour les patients séropositifs<br>**Format :** Tests sur place, services de traitement pré-VIH et/ou orientation vers une clinique de traitement du VIH	**Plan de l'étude :**<br>Essai non randomisé en groupes<br><br>**Sélection des sites :**<br>Spécifique<br>**Sélection des participants**<br>Consécutive	**Taille d'échantillon :** 882<br><br>**Âge :** 15-49 ans<br>**Sexe :** Féminin<br>**Suivi :** Recrutement initial en 1958 : 245 personnes exclues dont on sait qu'elles sont séropositives, 745 personnes sans historique complet des données de cohorte,<br>86 personnes ayant des données complètes manquantes sur toutes les variables potentiellement perturbantes
Criniti et al., 2011 [27]	**Lieu :**<br>É.-U./zone urbaine/clinique de PF financée par le Titre X<br><br>**Groupe cible :**<br>Patientes du planning familial	**Années de programme :** 2007-2009<br>**Années d'évaluation :** 2007-2009<br>**Nom :** N/A<br>**Intervention :** Renforcement des capacités du personnel médical des cliniques pour effectuer des tests de dépistage routinier rapides et non ciblés du VIH<br>**Format :** Test sur place et orientation vers une clinique prénatale spécialisée dans le VIH au sein du centre de PF	**Plan de l'étude :**<br>Étude de cohorte rétrospective<br><br>**Sélection du centre :** NR<br><br>**Sélection des participants**<br>Consécutive	**Taille d'échantillon :** NR (échantillon global de dossiers de patients NR ; population d'environ 9000 patients/an)<br><br>**Âge :** 15-49 ans<br>**Sexe :** Féminin<br>**Suivi :** N/A
Kimani et al., 2015 [28]	**Lieu :**<br>Kenya/établissements de santé ruraux et périurbains/publics (centres de santé, dispensaires, hôpitaux)<br><br>**Groupe cible :**<br>Femmes en période post-partum de 15 à 49 ans	**Années de programme :** 2010-2012<br>**Années d'évaluation :** 2010-2012<br>**Nom :** Integra Initiative<br>**Intervention :** Services VIH et PF intégrés dans les soins postnataux par rapport aux services indépendants<br>**Format :** Tests et conseils sur place	**Plan de l'étude :**<br>Essai non randomisé en groupes<br><br>**Sélection des sites :** Spécifique<br><br>**Sélection des participants** NR	**Taille d'échantillon :** 1693 (815 interventions, 878 comparaisons)<br><br>**Âge :** 15-49 ans<br>**Sexe :** Féminin<br>**Suivi :** 71 %<br>(573 interventions, 631 comparaisons)
Liambila et al., 2009 [29]	**Lieu :**<br>Kenya/NR/Planning familial avec conseil et dépistage proposés par le prestataire (SDV intégré), hôpitaux, centres de santé et dispensaires du secteur public<br><br>**Groupe cible :**<br>Patientes du planning familial	**Années de programme :** 2005-2007<br>**Années d'évaluation :** 2006-2007<br>**Nom :** N/A<br>**Intervention :** Planning familial avec conseil et dépistage proposés par le prestataire (SDV intégré)<br>**Format :** Tests et conseils sur place	**Plan de l'étude :**<br>Essai non randomisé en groupes<br><br>**Sélection des sites :** Spécifique<br><br>**Sélection des participants**<br>Consécutive	**Taille d'échantillon :** 1058<br><br>**Âge :** La plupart étaient âgés d'environ 30 ans<br>**Sexe :** Féminin<br>**Suivi :** N/A

SDV : Services de dépistage du VIH. PF : Planning familial. SMI : Service de santé maternelle et infantile. IST : Infection sexuellement transmissible. ES : Écart type. NR : Non rapporté. N/A : Non applicable.

Tableau 2. Rigueur de l'étude

Étude	Le plan d'étude inclut des données pré/post-intervention	Le plan d'étude inclut un groupe témoin ou de comparaison	Le plan d'étude inclut une cohorte	Groupes de comparaison équivalents au début en matière de données socio-démographiques	Groupes de comparaison équivalents au début en matière de critères de mesure des résultats	Affectation aléatoire (groupe ou individu) à l'intervention	Participants choisis au hasard pour évaluation	Contrôle des facteurs de confusion potentiels	Taux de suivi >=75 %
	Oui	Oui	Non	Non	Non	Non	Oui*	Oui**	N/A
	Non	Oui	Non	Oui	N/A	Oui	Oui	Non	N/A
	Non	Oui	Oui	Non	Non	Non	Oui*	Oui	Non
	Oui	Non	Non	N/A	N/A	Non	Oui*	Non	N/A
	Oui	Oui	Oui	Oui	Non	Non	Non	Oui	Non
	Oui	Oui	Non	Non	Non	Non	Oui*	Non	N/A

*Sélection d'échantillonnage/de recensement consécutive
**Pour les facteurs de confusion limités : nombre de patients par site, intégration au début, zones rurales/urbaines

Tableau 3. Résumé des principaux résultats obtenus.

<table>
<tr><th></th><th colspan="2">Catégorie de résultat de la question PICO</th></tr>
<tr><th>Étude</th><th>1) Adoption, conseil ou offre de services de dépistage du VIH</th><th>5) Satisfaction/perception de la qualité des services par les patients</th></tr>
<tr><td></td><td>
<table>
<tr><td></td><td>Sites de contrôle (n=4)</td><td>Sites d'intervention (n=4)</td></tr>
<tr><td colspan="3">Proportion de visites où les femmes ont bénéficié d'un conseil et d'un dépistage du VIH</td></tr>
<tr><td>2009</td><td>5 %-30 %</td><td>3 %-27 %</td></tr>
<tr><td>2010</td><td>2 %-14 %</td><td>8 %-16 %</td></tr>
<tr><td>2012</td><td>6 %-58 %</td><td>3 %-15 %</td></tr>
<tr><td colspan="3">Proportion de visites où les femmes ont reçu des services de VIH/d'IST et des services de SMI</td></tr>
<tr><td>2009</td><td>11 %-49 %</td><td>9 %-33 %</td></tr>
<tr><td>2010</td><td>3 %-27 %</td><td>2 %-21 %</td></tr>
<tr><td>2012</td><td>14 %-44 %</td><td>10 %-17 %</td></tr>
</table>
</td><td>Non mesuré</td></tr>
<tr><td></td><td>
<table>
<tr><td></td><td>Groupe de contrôle (n=119)</td><td>Intervention (n=137)</td><td>valeur p</td></tr>
<tr><td>A déjà subi un test de dépistage du VIH, n (%)</td><td>113 (94,96 %)</td><td>136 (99,27 %)</td><td>0,002</td></tr>
<tr><td>Nombre de tests au cours des 12 derniers mois, n (%)</td><td></td><td></td><td>0,043</td></tr>
<tr><td>0</td><td>22 (18,49 %)</td><td>10 (7,35 %)</td><td></td></tr>
<tr><td>1</td><td>20 (16,81 %)</td><td>20 (14,71 %)</td><td></td></tr>
<tr><td>2</td><td>31 (26,05 %)</td><td>28 (20,59 %)</td><td></td></tr>
<tr><td>3</td><td>34 (28,57 %)</td><td>44 (32,35 %)</td><td></td></tr>
<tr><td>>4</td><td>12 (10,08 %)</td><td>34 (25,00 %)</td><td></td></tr>
</table>
</td><td>Non mesuré</td></tr>
</table>

<table>
<tr>
<td></td>
<td>
Proportion de personnes ayant déclaré avoir subi un test de dépistage du VIH depuis le dernier entretien
<table>
<tr><th></th><th>Groupe d'intervention (n=439)</th><th>Groupe de comparaison (n=443)</th></tr>
<tr><td>R0 (immédiatement après l'intervention)</td><td>8,4</td><td>47,6</td></tr>
<tr><td>R1 (+6 mois)</td><td>44,7</td><td>51,5</td></tr>
<tr><td>R2 (+18 mois)</td><td>64,0</td><td>66,4</td></tr>
<tr><td>R3 (+24 mois)</td><td>71,8</td><td>60,7</td></tr>
</table>
Pourcentage de femmes atteignant les objectifs de dépistage du VIH (minimum de deux tests, un test par an) au cours de la cohorte de deux ans, par différents groupes d'exposition

Davantage de femmes dans le groupe de comparaison du VIH (73 %) ont atteint l'objectif de dépistage du VIH par rapport au groupe d'intervention (65 %) (p<0,05).
Les femmes ayant reçu des services intégrés au départ, quel que soit le groupe, étaient plus susceptibles de recevoir un minimum de deux tests après R0 (71 %) par rapport à celles qui n'en avaient pas (61 %) (p<0,01).
Les femmes présentant l'exposition cumulée la plus élevée aux services intégrés étaient plus susceptibles d'avoir satisfait à l'exigence de test (77 %) par rapport au groupe à score moyen (71 %) et au groupe à score faible (60 %) (p<0,001).

</td>
<td>

Les femmes des sites d’intervention étaient plus susceptibles d’être très satisfaites des services (30 % contre 27 %)
Les femmes des sites d’intervention étaient plus susceptibles d’attendre plus de 30 minutes pour obtenir des services (57 % contre 0,2 %)
Les femmes des sites d’intervention étaient moins susceptibles d’avoir déboursé des frais pour leurs services (83 % contre 93 %).

</td>
</tr>
<tr>
<td></td>
<td>
<table>
<tr><th></th><th>Avant le dépistage rapide du VIH (avant 2003)</th><th>Échantillon-test de VIH défini (2003-2007)</th><th>Intégration complète dans le flux de services des cliniques (2007-2009)</th><th></th></tr>
<tr><td>Taux d'acceptation des tests</td><td>Indisponible</td><td>76 %</td><td>89 %</td><td></td></tr>
<tr><td>Patients avec un test documenté du VIH dans le dossier médical au cours des 12 derniers mois</td><td>34 %</td><td>65 %</td><td>71 %</td><td></td></tr>
<tr><td>Nombre moyen de tests effectués par mois</td><td>Indisponible</td><td>70</td><td>87,9</td><td></td></tr>
</table>
</td>
<td>Non mesuré</td>
</tr>
<tr>
<td></td>
<td>
Adoption des conseils et dépistages proposés par le prestataire
<table>
<tr><th></th><th>Groupe de contrôle n/N (%)</th><th>Groupe d'intervention n/N (%)</th></tr>
<tr><td>Au début</td><td>87/878 (9,9)</td><td>125/815 (15,3)</td></tr>
<tr><td>Suivi à 15 mois</td><td>104/631 (29,6)</td><td>157/573 (46,6)</td></tr>
</table>
aOR pour site d'intervention comparativement au groupe témoin : 1,6, (IC 95 % : 1,2-2,2) (p<0,01)
</td>
<td>Non mesuré</td>
</tr>
<tr>
<td></td>
<td>
<table>
<tr><th></th><th>Modèle de test % (N)</th><th>Modèle d'orientation % (N)</th></tr>
<tr><td colspan="3">Proportion de nouveaux patients testés après l'introduction de l'intervention</td></tr>
<tr><td>Nouveaux patients à qui on a proposé un test de dépistage du VIH*</td><td>74 (27)</td><td>34 (50)</td></tr>
<tr><td>Si proposé, nouveaux patients ayant accepté le test de dépistage du VIH</td><td>50 (20)</td><td>65 (17)</td></tr>
<tr><td>Proportion de tous les nouveaux clients ayant été dépistés*</td><td>37 (27)</td><td>22 (50)</td></tr>
<tr><td colspan="3">Proportion de patients existants dépistés lors d'une nouvelle visite</td></tr>
<tr><td>Patients existants à qui on a proposé un test de dépistage du VIH*</td><td>56 (183)</td><td>27 (259)</td></tr>
<tr><td>Si proposé, patients existants ayant accepté le test de dépistage du VIH</td><td>61 (103)</td><td>72 (69)</td></tr>
<tr><td>Proportion de tous les patients existants dépistés*</td><td>34 (183)</td><td>19 (259)</td></tr>
<tr><td colspan="3">Proportion de tous les patients dépistés</td></tr>
<tr><td>Proportion de tous les patients nouveaux/existants dépistés*</td><td>35 (210)</td><td>20 (309)</td></tr>
</table>
*importante à p<0,01
</td>
<td>Non mesuré</td>
</tr>
</table>

**Remarque : ce tableau inclut uniquement les données de résultat répondant à la question PICO en comparant les services de dépistage du VIH intégrés aux services de planning familial aux services non intégrés. Aucune des études n'a rapporté les résultats PICO n° 2) nouveaux cas de VIH identifiés, n° 3) liens avec les soins et le traitement du VIH, n° 4) utilisation de la double méthode et n° 6) connaissance et attitudes du prestataire concernant l'intégration du SDV
