# Supplementary material for: Integration of HIV testing services into family planning services: a systematic review
Source: Reprod Health. 2019 May 29;16(Suppl 1):61. doi: 10.1186/s12978-019-0714-9 (PMC6538541; doi:10.1186/s12978-019-0714-9)
Supplement: Supplementary file 2 — Translation of the abstract of this article into Portuguese. (PDF 97 kb) [file 12978_2019_714_MOESM2_ESM.pdf]

Reproductive Health 2019, 16 (Suppl 1) [translated from the original publication]

Integração dos serviços de despistagem do VIH nos serviços de planeamento familiar: uma revisão sistemática

Manjulaa Narasimhan1*, Ping Teresa Yeh[2], Sabina Haberlen[3], Charlotte E. Warren[4], Caitlin E. Kennedy[2]

[1]Department of Reproductive Health and Research and UNDP/UNFPA/UNICEF/WHO/World Bank Special Programme, World Health Organization, Geneva, Switzerland
[2]Department of International Health, Johns Hopkins Bloomberg School of Public Health, Baltimore, Maryland, USA
[3]Department of Epidemiology, Johns Hopkins Bloomberg School of Public Health, Baltimore, Maryland, USA
[4]Population Council, Washington, District of Columbia, USA

*Autor correspondente: Manjulaa Narasimhan: narasimhanm@who.int
E-mail dos autores:
Ping Teresa Yeh: teresa.yeh@jhu.edu
Sabina Haberlen: shaberlen@jhu.edu
Charlotte E. Warren: cwarren@popcouncil.org
Caitlin E. Kennedy: caitlinkennedy@jhu.edu

Resumo

Introdução: Apesar de existir um interesse significativo em integrar os serviços de saúde sexual e reprodutiva (SSR) nos serviços de VIH, a articulação com outros serviços foi negligenciada. Quando as mulheres e raparigas correm o risco de contrair VIH, a oferta de serviços de despistagem do VIH durante as visitas aos serviços de planeamento familiar (PF) proporciona oportunidades importantes para responder, em simultâneo, às necessidades ao nível de VIH e gravidez indesejada.

Métodos: Foi efetuada uma revisão sistemática de estudos que compara os serviços de PF com e sem serviços de despistagem do VIH integrados ou serviços com um nível inferior de integração (por exemplo, serviços de referência vs. serviços no local), para os seguintes resultados: acesso/aconselhamento/oferta de serviços de despistagem do VIH, novos casos de VIH identificados, articulação com cuidados de saúde e tratamento do VIH, utilização do método duplo, satisfação do cliente e qualidade do serviço, bem como o conhecimento, as

competências e a atitude dos prestadores ao nível da integração dos serviços de despistagem do VIH. Foram pesquisadas três bases de dados online e incluídos estudos publicados numa revista científica anterior à data de pesquisa de 20 de junho de 2017.

Resultados: Das 530 referências, seis estudos cumpriram os critérios de inclusão. Foram realizados três estudos no Quénia, um no Uganda, um na Suazilândia e um nos EUA. A maior parte dos estudos foi realizada em clínicas de PF. Três estudos têm autoria da Integra Initiative. O nível de rigor global foi moderado com um ensaio de grupo aleatório. O acesso aos serviços de despistagem do VIH foi tendencionalmente superior em locais integrados em comparação com locais pré-integrados, inclusive nas análises ajustadas, apesar dos resultados variarem ligeiramente nos estudos. Um estudo constatou que as mulheres em locais integrados têm uma maior probabilidade de obter um nível superior de satisfação com os serviços prestados , mas que tiveram tempos de espera mais prolongados. Outro estudo verificou um ligeiro aumento na seropositividade do VIH nos testes de pacientes do sexo feminino após integração total, em comparação com um sistema de teste do VIH dedicado. Nenhum estudo analisou comparativamente a articulação aos cuidados de saúde e tratamento do VIH, utilização do método duplo ou conhecimento/atitude dos prestadores.

Conclusões: O progresso e sucesso global na consecução dos objetivos em matéria de SSR e VIH depende do progresso na África Subsariana, onde as mulheres têm a taxa mais elevada de gravidez indesejada e infeções sexualmente transmissíveis, incluindo o VIH. Embora a base factual seja limitada, sugere que a integração dos serviços de despistagem do VIH nos serviços de PF é viável e que pode ter resultados positivos comuns. O sucesso e a expansão desta abordagem irão depender das necessidades da população e dos fatores do sistema de saúde.

Palavras-chave: Integração de serviços, Planeamento Familiar, VIH, Saúde Sexual e

Reprodutiva, Acesso

Sobre este suplemento

Este resumo foi publicado como parte da revista científica *Reproductive Health*, Volume 16, Suplemento 1, 2019: Integração Eficaz dos Serviços de Saúde Sexual e Reprodutiva e de Prevenção, Cuidados e Tratamento do VIH na África Subsariana: Onde estão as provas da implementação do programa?
O suplemento foi publicado como uma colaboração entre as revistas científicas *Reproductive Health* e *BMC Public Health.* O conteúdo integral, incluindo as versões em francês, português e inglês, estão disponíveis online:
https://bmcpublichealth.biomedcentral.com/articles/supplements/volume-19-supplement-1
e
https://reproductive-health-journal.biomedcentral.com/articles/supplements/volume-16-supplement-1
